# Mammalian Eps15 homology domain 1 potentiates angiogenesis of non-small cell lung cancer by regulating β2AR signaling

**DOI:** 10.1186/s13046-019-1162-7

**Published:** 2019-04-25

**Authors:** Ting Wang, Ying Xing, Qingwei Meng, Hailing Lu, Wei Liu, Shi Yan, Yang Song, Xinyuan Xu, Jian Huang, Yue Cui, Dexin Jia, Li Cai

**Affiliations:** 1 The Fourth Department of Medical Oncology, Harbin Medical University Cancer Hospital, 150 Haping Road, Harbin, 150040 China; 2 The Sixth Department of Medical Oncology, Harbin Medical University Cancer Hospital, 150 Haping Road, Harbin, 150040 China; 3 Department of Orthopedic Surgery, The Second Affiliated Hospital of Harbin Medical University, Xuefu Road 246, Harbin, 150081 China

**Keywords:** EHD1, NSCLC, Angiogenesis, β2AR signaling, Endocytosis

## Abstract

**Background:**

Non-small cell lung cancer (NSCLC) is a devastating disease with a heterogeneous prognosis, and the molecular mechanisms underlying tumor progression remain elusive. Mammalian Eps15 homology domain 1 (EHD1) plays a promotive role in tumor progression, but its role in cancer angiogenesis remains unknown. This study thus explored the role of EHD1 in angiogenesis in NSCLC.

**Methods:**

The changes in angiogenesis were evaluated through human umbilical vein endothelial cell (HUVEC) proliferation, migration and tube formation assays. The impact of EHD1 on β2-adrenoceptor (β2AR) signaling was evaluated by Western blotting, quantitative real-time polymerase chain reaction (qRT-PCR) analysis, and enzyme-linked immunosorbent assay (ELISA). The interaction between EHD1 and β2AR was confirmed by immunofluorescence (IF) and coimmunoprecipitation (Co-IP) experiments, and confocal microscopy immunofluorescence studies revealed that β2AR colocalized with the recycling endosome marker Rab11, which indicated β2AR endocytosis. Xenograft tumor models were used to investigate the role of EHD1 in NSCLC tumor growth.

**Results:**

The microarray analysis revealed that EHD1 was significantly correlated with tumor angiogenesis, and loss- and gain-of-function experiments demonstrated that EHD1 potentiates HUVEC proliferation, migration and tube formation. EHD1 knockdown inhibited β2AR signaling activity, and EHD1 upregulation promoted vascular endothelial growth factor A (VEGFA) and β2AR expression. Interestingly, EHD1 interacted with β2AR and played a novel and critical role in β2AR endocytic recycling to prevent receptor degradation. Aberrant VEGFA or β2AR expression significantly affected EHD1-mediated tumor angiogenesis. The proangiogenic role of EHD1 was confirmed in xenograft tumor models, and immunohistochemistry (IHC) analysis confirmed that EHD1 expression was positively correlated with VEGFA expression, microvessel density (MVD) and β2AR expression in patient specimens.

**Conclusion:**

Collectively, the data obtained in this study suggest that EHD1 plays a critical role in NSCLC angiogenesis via β2AR signaling and highlight a potential target for antiangiogenic therapy.

**Electronic supplementary material:**

The online version of this article (10.1186/s13046-019-1162-7) contains supplementary material, which is available to authorized users.

## Background

Lung cancer is the leading type of cancer worldwide [1], and non-small cell lung cancer (NSCLC) is the most frequent type of lung cancer, accounting for 87% of lung cancer cases, and has a 5-year survival rate of less than 17% [2]. Angiogenesis, the process of new blood vessel formation, is crucial for tumor growth, but the molecular mechanisms of angiogenesis in NSCLC remain unclear [3, 4]. To improve patient outcome, new genes related to angiogenesis must be identified, and the molecular mechanisms underlying tumor neovascularization must be elucidated.

C-terminal Eps15-homology (EH) domain-containing protein (EHD1) regulates cellular receptor recycling from the endocytic recycling compartment to the plasma membrane [5, 6]. Structurally, EHD1 has a single EH domain at its C terminus, a central coiled-coil region involved in oligomerization, and an N-terminal regulatory region that binds to nucleotides [6, 7]. Importantly, C-terminal EHD proteins play an important role in regulating the transport of receptors, such as epithelial growth factor receptor [8], insulin-like growth factor receptor [9] and colony-stimulating factor-1 receptor [10]. An accumulating body of evidence implicates EHD1 in the development and progression of multiple types of cancers, such as breast cancer [8], thyroid cancer [11], ovarian cancer [12] and lung cancer [13]. Over the past five years, we have revealed that EHD1 overexpression in NSCLC predicts poor prognosis for patients and that EHD1 might play a pivotal role in tumor metastasis, stemness, chemotherapy resistance and epidermal growth factor receptor (EGFR)-tyrosine kinase inhibitor (TKI) resistance [13–15]. However, the involvement of EHD1 in tumor angiogenesis is unknown.

β2-adrenoceptors (β2ARs), the best characterized β-adrenergic receptor proteins thus far, are prototypic and ubiquitous cell-surface proteins known as G protein-coupled receptors or seven-transmembrane receptors [16]. In cardiac disease, the activation of cardiomyocyte β2AR by catecholamines leads to pathological responses [17]. Recently, focus has turned toward understanding the regulatory role of β2AR in tumourigenesis [18]. Studies have shown that β2AR signaling stimulates pathological angiogenesis, which is an essential strategy used by tumor cells to obtain various nutrients and favors tumor growth and progression [19, 20]. Mechanistically, ligand-β2AR-cAMP-protein kinase A (PKA)-driven angiogenic growth factors are produced by endothelial cells [19]. Among these factors, vascular endothelial growth factors (VEGFs), particularly VEGFA, act as potent endothelial mitogens to induce a rapid and complete angiogenic response and organize vascular patterns [21]. β2AR signaling plays a well-documented role in promoting cancer progression in various malignancies, such as lung cancer [18], breast cancer [20] and gastric cancer [22]. Therefore, further investigations are needed to better elucidate the β2AR signaling pathway and identify its upstream signal targets.

In this study, we examined the effect of EHD1 on tumor angiogenesis in NSCLC and found that EHD1 induces NSCLC angiogenesis by upregulating VEGFA expression. Furthermore, we demonstrated that EHD1 has a proangiogenic function through its regulation of the β2AR signaling pathway in NSCLC and revealed that EHD1 participates in β2AR endocytosis and recycling.

## Materials and methods

### Microarray processing and analysis

Detailed information on the microarray processing and analysis was described previously [13].

### Cell culture

The human NSCLC cell lines NCI-H1650, PC9, NCI-H1299, NCI-H827, NCI-H520, A549, NCI-H1975, PC14, NCI-H466, NCI-H2170 and NCI-H460 and human umbilical vein endothelial cells (HUVECs) were purchased from the American Type Culture Collection (ATCC, Manassas, VA, USA). The human NSCLC cell lines NCI-H1650, NCI-H1299, NCI-H827, NCI-H520, A549, NCI-H1975, PC14, NCI-H466, NCI-H2170 and NCI-H460 were maintained in 1640 medium supplemented with 10% fetal bovine serum (FBS, Gibco) and 1% penicillin/streptomycin (Gibco). The PC9 cells were cultured in Dulbecco’s modified Eagle’s medium (DMEM) supplemented with 10% FBS and 1% penicillin/streptomycin (Gibco). The HUVECs were incubated with Ham’s F-12 K supplemented with 100 μg/ml heparin (Sigma), 50 μg/ml endothelial cell growth supplement (BD Biosciences), 10% FBS (Gibco) and 1% penicillin/streptomycin (Gibco).

### Western blot analysis

Antibodies against the following proteins were used in this study: EHD1 (ab109311, Abcam, Cambridge, MA, USA), VEGFA (ab1316, Abcam, Cambridge, MA, USA), β2AR (ab182136, Abcam, Cambridge, MA, USA), β2AR (sc-271,322, Santa Cruz), β-actin (TA-09, ZSGB-Bio, China) and glyceraldehyde-3-phosphate dehydrogenase (GAPDH; TA-08, ZSGB-Bio, China). The photodensity of Western blot bands was quantified using ImageJ software (U.S. National Institutes of Health, USA).

### Conditioned medium

The A549 and NCI-H1650 cells were grown to 70–80% confluence and then incubated in serum-free DMEM for 16 h or in serum-free DMEM with isoprenaline hydrochloride (10 μm) (HY-B0468, MedChem Express) or ICI118,551 (ICI, 10 μm) (HY-13951, MedChem Express) for 16 h under the same conditions. Conditioned media (CMs) were collected, centrifuged at 2000 rpm and 4 °C for 10 min, filtered, and stored at − 70 °C.

### HUVEC proliferation assay

HUVECs were seeded in 96-well plates at a density of 4000 cells per well and incubated with the corresponding CM, CM with VEGFA or CM with apatinib for 24 h, 48 h and 72 h. The cell viability rate was evaluated using the Cell Counting Kit-8 (Dojindo Molecular Technologies, Kumamoto, Japan). The optical density (OD) value was measured at 450 nm.

### HUVEC migration assay

HUVECs were grown to 70% confluence and serum-starved overnight. As previously described [23], 5 × 10^4^ HUVECs were trypsinized, suspended in serum-free medium and seeded in the upper chamber insert (#3422 Costar, Corning, NY, USA), and CM (800 μl) was added to the lower chamber. After 24 h, the migratory cells on the lower surface of the membrane were fixed with methanol and stained with crystal violet for 30 min. The stained cells were observed and captured using a light microscope (Olympus), and the numbers of migratory cells in three random fields were quantified.

### HUVEC tube formation assay

HUVECs (8 × 10^4^) were starved overnight, incubated with the corresponding NSCLC CM (200 μl) and seeded in a 24-well plate coated with Matrigel (200 μl/well, BD Biosciences). After 6 h of incubation at 37 °C with 5% CO_2_, the capillary tube structure was observed and captured using a light microscope (Olympus). The number of tubes was counted and compared between different groups [24]. Each condition was assessed in triplicate.

### The Cancer genome atlas (TCGA) and Cancer cell line encyclopedia (CCLE) data analysis

Pan-cancer and lung cancer patient data were obtained from the TCGA database. Data on the expression of EHD1 and β2AR in NSCLC cells were downloaded from the CCLE database.

### Enzyme-linked immunosorbent assay (ELISA)

The VEGFA concentrations in the CMs were detected using a human VEGFA ELISA kit (USCN Life Science Inc., Wuhan, China). The measurements were performed in accordance to the instructions provided by the manufacturer and acquired with a microplate reader (BioTek, Winooski, VT, USA) at 450 nm.

### qRT-PCR

A qRT-PCR analysis was performed as previously described [25]. DNA was reacted with Fast SYBR Green Master Mix (Applied Biosystems) using the following primers: 5′-CCACAAGCTGGACATCTCCGATGAG-3′ (forward) and 5′-GGGACCAGAAGGAGCCGATGTAGAC 3′ (reverse) for EHD1; 5′-GATGGTGTGGAATTGTGTCAG-3′ (forward) and 5′-GCAGGTCTCATTGGCATAGC-3′ (reverse) for β2AR; 5′-GAAGTGGTGAAGTTCATGGATGTCT-3′ (forward) and 5′- ATGGTGATGTTGGACTCCTCAGTG-3′ (reverse) for VEGFA, and 5′-CTTAGTTGCGTTACACCCTTTCTTG-3′ (forward) and 5′-CTGTCACCTTCACCGTTCCAGTTT-3′ (reverse) for β-actin.

### Coculture experiments

The A549 and NCI-H1650 cells were seeded into six-well coculture plates, grown to 70–80% confluence, and incubated in low-serum 1% FBS-containing DMEM medium for 24 h. A total of 5 × 10^4^ HUVECs were seeded in each cell culture insert (pore size of 0.4 μm, BD Biosciences, CA, USA) for 24 h. The HUVECs in these inserts were cocultured for 48 h with the pretreated A549 or NCI-H1650 cells, which were placed in the plate of the lower chamber, and then collected for Western blot analysis.

### Immunoprecipitation (IP) assay

IP assays were conducted with the Crosslink Magnetic IP/Co-IP kit (Thermo, Rockford, IL, USA). The measurements were performed based on the instructions provided by the manufacturer, as previously described [25].

### Immunofluorescence

Measurements of immunofluorescence were performed as previously described [25]. The cells were incubated with the indicated primary antibodies against β2AR (1:100 dilution, sc-271,322, Santa Cruz) and Rab11 (1:100 dilution, #5589, Cell Signaling Technology) overnight at 4 °C and then with Alexa Fluor 488-conjugated anti-mouse IgG (sc-516,140, Santa Cruz) or Alexa Fluor 647-conjugated anti-rabbit IgG (ab150075, Abcam, Cambridge, MA, USA) in the dark at room temperature for 1 h. The stained cells were observed and captured using a laser-scanning confocal microscope (LSM510, Carl Zeiss, Inc.).

### Xenograft models

Female, 4–5-week-old BALB/c nude mice were obtained from Beijing Vital River Laboratory Animal Technology Co., Ltd., and bred at the Animal Center of the Second Affiliated Hospital of Harbin Medical University. The BALB/c nude mice were randomly divided into experimental groups (*n* = 5/group), and 5 × 10^6^ cells were injected subcutaneously into the alar skin of the nude mice. Seven days after implantation, the mice were randomly divided into two subgroups. The initial luciferase signals and tumor volume were then measured, and the mice were then administered apatinib (200 mg/kg) or PBS once daily by oral gavage. Subsequently, the tumor volume was monitored with Vernier calipers every week for 4 weeks and calculated using the eq. (L × W^2^)/2, where L is the length and W is the width. At day 28, the initial luciferase signals were measured, the animals were sacrificed, and the tumor tissues were removed. Part of the tumor tissues was frozen at − 80 °C for Western blot assay, and the remaining tissue was fixed and paraffin-embedded for immunohistochemical analysis.

### Immunohistochemistry (IHC)

The detailed experimental immunohistochemical procedures were described previously [25]. The protein expression levels of EHD1, β2AR and VEGFA were assessed by IHC with the corresponding anti-EHD1 (dilution 1:25, ab109311, Abcam, Cambridge, MA, USA), anti-β2AR (dilution 1:40, AF6117, Affinity Biosciences, China) and anti-VEGFA antibodies (dilution 1:100, ab 52,917, Abcam, Cambridge, MA, USA), respectively. Angiogenesis was evaluated through IHC staining of human and mouse tumor tissues with the anti-CD31 antibody (dilution 1:50, ab28364, Cambridge, MA, USA). The levels of EHD1, β2AR and VEGFA staining were scored based on previously described criteria [25]. The microvessel density (MVD) in tumor samples was assessed based on CD31 staining. The MVD value was obtained as the median from the values obtained for three vascularized areas at 200× magnification. Using the average MVD in human tissues as the threshold, the tissues were split into a low group (*n* ≤ 9) and a high group based on their MVD status (*n* > 9).

### Statistical analysis

All statistical analyses were performed with SPSS 22.0 and GraphPad Prism software. The data are expressed as the means ± standard deviations (SDs). The differences between two groups were analyzed with Student’s tests and the χ2 test. The survival analysis was performed using Kaplan-Meier analysis and log-rank tests. A two-tailed *p* value of < 0.05 was considered significant.

## Results

### EHD1 expression predicts NSCLC and pan-cancer prognosis

IHC analyses revealed that increased EHD1 expression was correlated with advanced pT classification and advanced pTNM stage in patients from Harbin Medical University Cancer Center (HMUCC) (Additional file 1: Table S1). We subsequently performed Kaplan-Meier analyses and found that high EHD1 expression predicts a poor prognosis in terms of both overall survival (OS) and disease-free survival (DFS) (Additional file 2: Figure S1a-b). Our results based on the TCGA database, which were mainly analyzed using the web-based tools in Gene Expression Profiling Interactive Analysis (GEPIA, http://gepia.cancer-pku.cn/) [26], showed that high EHD1 expression was an unfavorable predictor for NSCLC patients (Additional file 2: Figure S1c-d). Moreover, using data from 10,704 tumors in the TCGA database across 26 disease sites, we evaluated the predictive value of EHD1 gene expression for the prognosis of cancer patients. As shown in Additional file 2: Figure S1e and f, high EHD1 expression was a predictor of poor OS and progression-free interval in pan-cancer.

### EHD1 induces angiogenesis in NSCLC

**Fig. 1:**
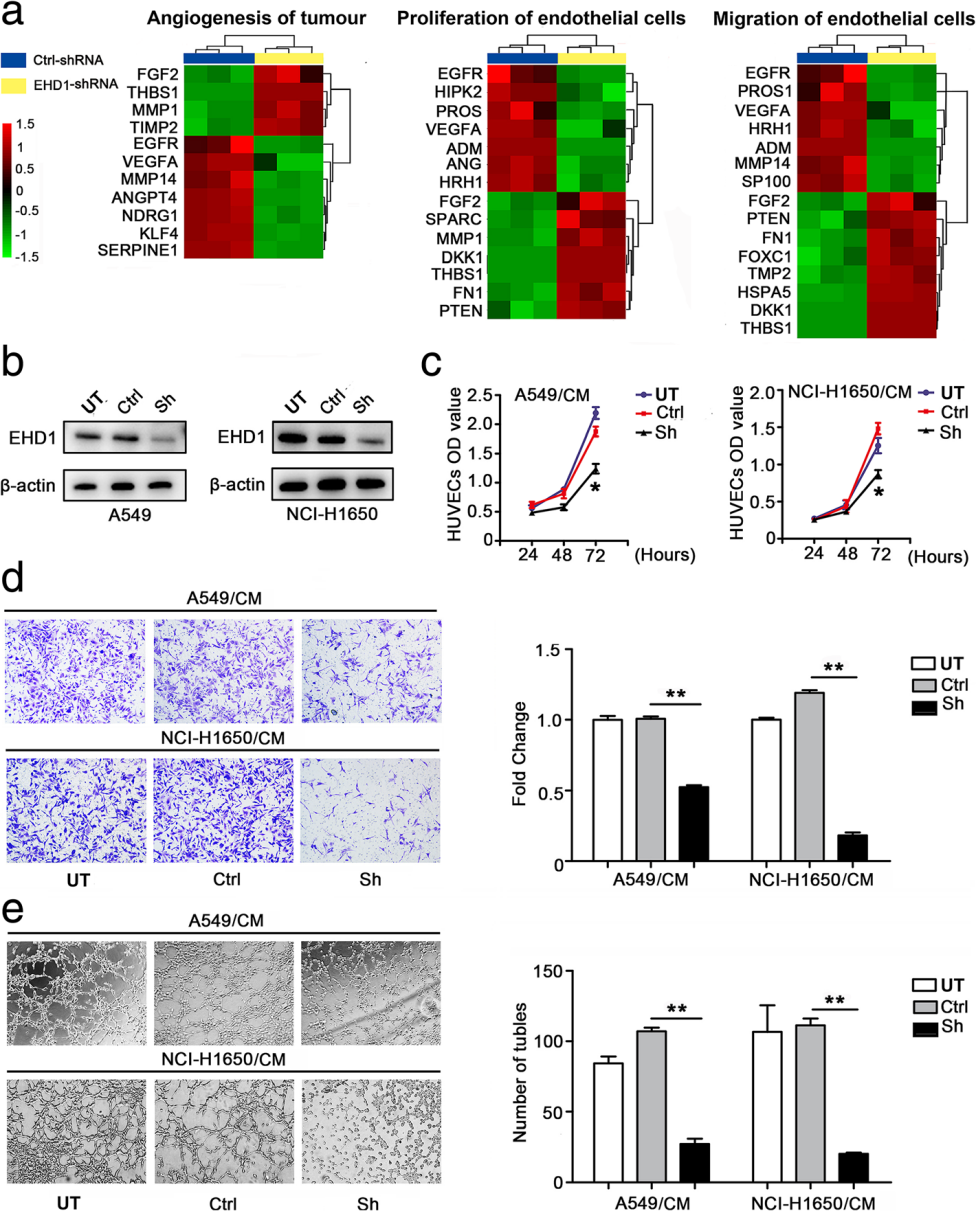
EHD1 induces angiogenesis in NSCLC. **a** Microarray analysis demonstrating the positive correlation between EHD1 and tumour angiogenesis and vascular endothelial cell proliferation and migration. The pseudocolor represents the intensity scale for the EHD1 ShRNA vector versus the control, as calculated by log2 transformation. **b** Western blot analysis of EHD1 expression in A549 and NCI-H1650 cells after EHD1 knockdown. β-actin served as the loading control. **c** The viability of HUVECs was detected by CCK8 assay. HUVECs were incubated in 96-well plates with CMs from A549 and NCI-H1650 cells. **d** CMs were added to the lower chamber, and HUVECs were seeded on the upper chamber. After 24 h of incubation, HUVEC migration was assessed by counting the cells on the lower surface of the membrane; from left to right, the lanes show UT, Ctrl and ShEHD1. Scale bar, 100 μm. **e** HUVECs were incubated in 48-well plates with CMs from A549 and NCI-H1650 cells, and their tube formation was evaluated based on the number of tubes per field. *p < 0.05; **p < 0.01

A microarray analysis performed using the Affymetrix Human Gene 1.0 ST platform revealed a significant positive correlation between EHD1 and tumor angiogenesis and vascular endothelial cell proliferation and migration (Fig. 1a, Additional file 3: Table S2, Additional file 4: Table S3 and Additional file 5: Table S4). To test the effect of EHD1 on in vitro angiogenesis, A549 and NCI-H1650 cells were selected as a “loss-of-function” model due to their high expression of EHD1 [13]. We knocked down EHD1 expression in these NSCLC cells using shRNA targeting EHD1 (Fig. 1b). We then treated HUVECs with CM from untreated cells (UT), CM from control cells transfected with scrambled shRNA (Ctrl) or CM from EHD1-downregulated cells (Sh). The evaluation of vitro angiogenesis activity based on the proliferation, migration and tube formation abilities of endothelial cells has been previously described [23]. Compared with the CMs from UT and Ctrl, the CM from Sh resulted in decreased HUVEC proliferation, migration and tube formation abilities (Fig. 1c-e).

To further validate the role of EHD1 in NSCLC angiogenesis, we conducted a rescue expression experiment in which Sh were transfected with a vector encoding the human EHD1 gene (the resulting cells were designated Sh/R) or with an empty vector (control, the resulting cells were designated Sh/Ctrl) (Additional file 6: Figure S2a). Treatment with the CM from Sh/R enhanced the abilities of HUVECs to proliferate, migrate and form tubes compared with treatment with the CMs from Sh and Ctrl (Additional file 6: Figure S2b-d).

### EHD1 promotes angiogenesis in a VEGFA-dependent manner

**Fig. 2:**
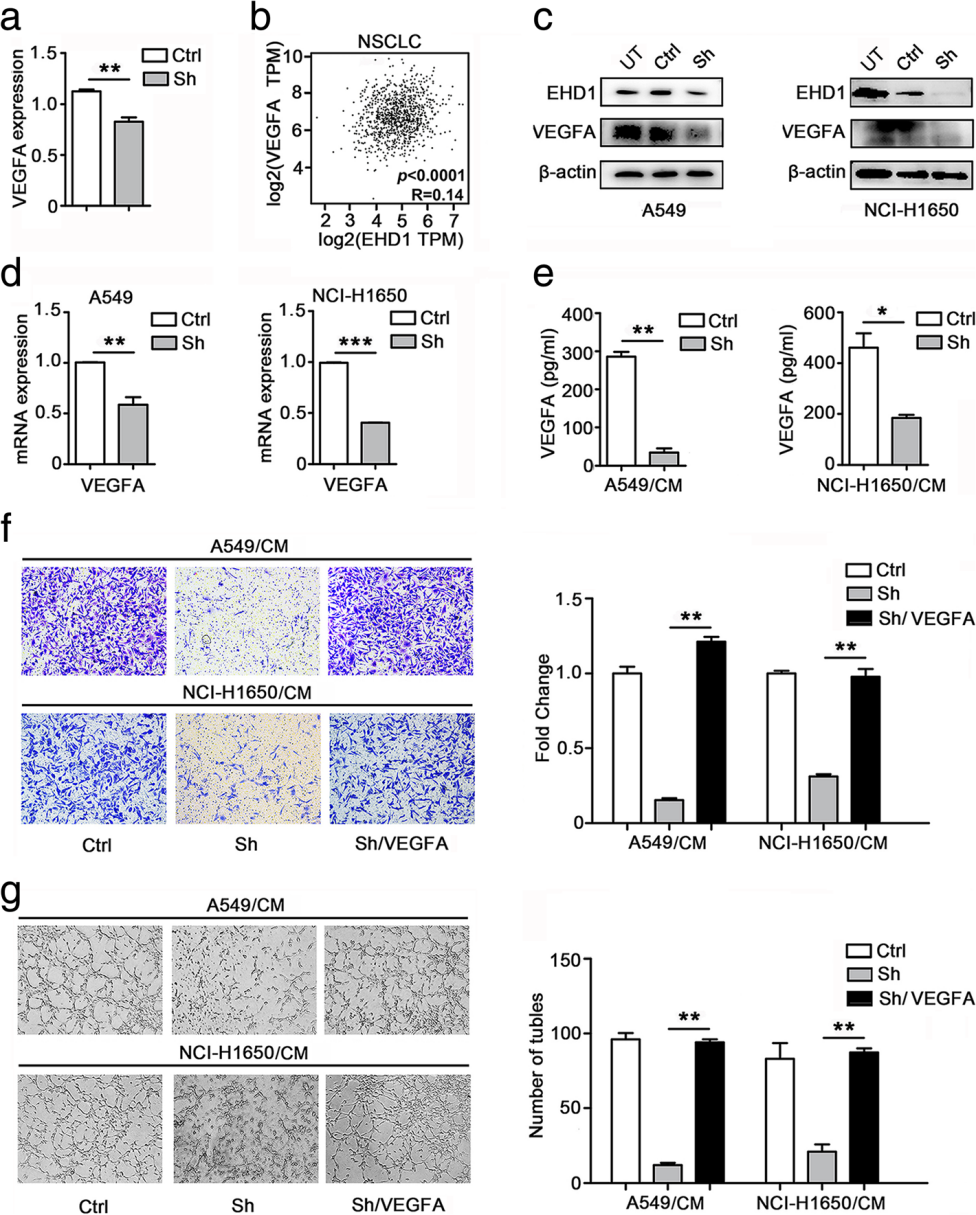
EHD1-induced angiogenic effects depend on VEGFA expression. **a** mRNA expression of VEGFA. **b** Correlation between EHD1 and VEGFA expression in the TCGA database (R = 0.14, *p* < 0.0001). **c** Western blot analysis of VEGFA expression in A549 and NCI-H1650 cells after EHD1 knockdown. **d** qRT-PCR analysis of VEGFA mRNA levels in A549 cells and NCI-H1650 cells. **e** A549 and NCI-H1650 cells were incubated overnight with serum-free 1640 medium, and their CMs were used to detect the level of VEGFA secretion; the lanes in the left and right show Ctrl and Sh, respectively. **f** CMs were added to the lower chamber, and HUVECs were seeded on the upper chamber. After 24 h of incubation, HUVEC migration was assessed by counting the cells on the lower surface of the membrane. Scale bar, 100 μm. **g** HUVECs were incubated in 48-well plates with CMs from A549 and NCI-H1650 cells, and their tube formation abilities were evaluated based on the number of tubes per field

VEGFA plays a critical role in angiogenesis [27]. The microarray data indicated that the mRNA levels of VEGFA were downregulated in the EHD1-knockdown NSCLC cells compared with the levels in the control cells (Fig. 2a, Additional file 3: Table S2, Additional file 4: Table S3 and Additional file 5: Table S4). Based on the TCGA database, we found a positive correlation between EHD1 and VEGFA expression (*p* < 0.0001, R = 0.14; Fig. 2b). A Western blot assay using antibodies targeting VEGFA revealed that the EHD1-knockdown cells showed less VEGFA protein than the control cells (Fig. 2c). As expected, the EHD1-knockdown cells showed less VEGFA mRNA expression than the control cells (Fig. 2d). As demonstrated by ELISA, the production of VEGFA was reduced in the NSCLC cells transfected with shRNA targeting EHD1 (Fig. 2e). To further investigate the involvement of VEGFA in EHD1-mediated angiogenesis in vitro, DMSO or VEGFA was added to the CM from Sh (Sh/CM), and subsequent in vitro HUVEC migration and tube formation assays revealed that the inhibitory effect of EHD1 knockdown on angiogenesis was reversed by VEGFA (Fig. 2f, g).

VEGFA promotes angiogenesis through activation of the phosphoinositide 3-kinase (PI3K)/Akt and Erk signaling pathways [28]. After coculture with Sh or Ctrl, we tested the expression of PI3K/Akt and Erk signaling molecules by Western blotting and demonstrated that p-AKT and p-Erk expression was significantly attenuated in HUVECs cocultured with Sh compared with their levels in HUVECs cocultured with Ctrl (Additional file 7: Figure S3).

Consistent with the above results, the reexpression of EHD1 in Sh increased the expression of VEGFA protein (Additional file 8: Figure S4a-b). Apatinib, a specific inhibitor of VEGFR2, completely abolished the EHD1-induced angiogenic effects (Additional file 8: Figure S4c, d). Together, these data show that the stimulation of cancer cell angiogenesis by EHD1 is highly dependent on VEGFA.

### EHD1 activates β2AR signaling in NSCLC

**Fig. 3:**
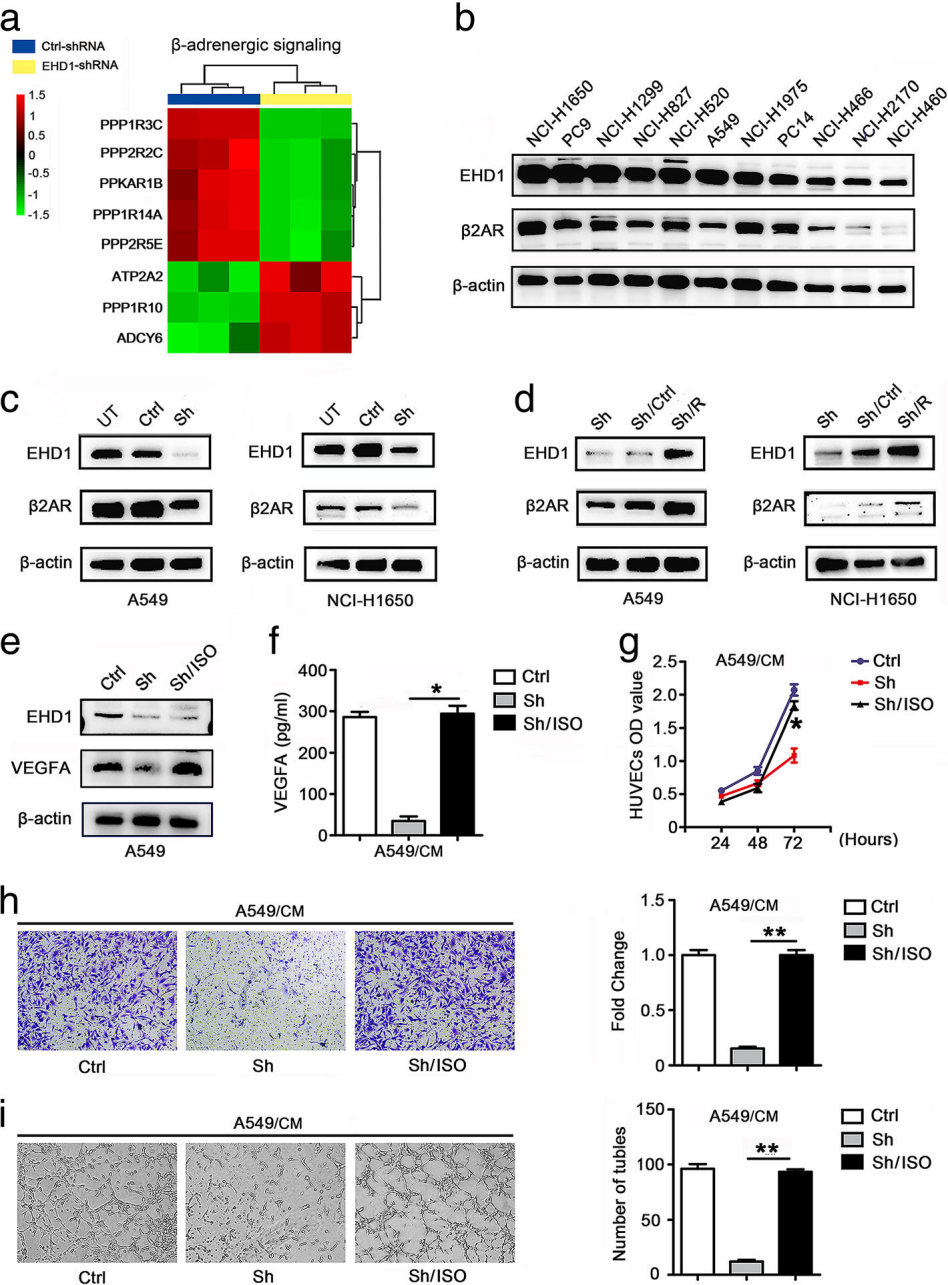
EHD1 induces VEGFA expression and angiogenesis in a β2AR-dependent manner in NSCLC. **a** Microarray analysis showed an obvious overlap between β2AR-dependent gene expression and EHD1-regulated gene expression. The pseudocolor represents the intensity scale for the EHD1 ShRNA vector versus the control, as calculated by log2 transformation. **b** Western blot of EHD1 expression in human lung cancer cells. **c** Western blot analysis of β2AR expression in A549 and NCI-H1650 cells after EHD1 knockdown. **d** Immunoblotting analysis of β2AR expression in A549 and NCI-H1650 cells after EHD1 reexpression. **e** A549 cells were transfected with vector or ShEHD1 and then treated with DMSO or ISO, and the EHD1 and VEGFA protein levels were assessed by immunoblotting. **f** A549 cells were incubated overnight with serum-free 1640 medium and corresponding reagents, and their CMs were used to test the level of VEGFA secretion by ELISA. **g** The viability of HUVECs was detected by CCK8 assay. HUVECs in 96-well plates were incubated with CMs from A549 cells. **h** CMs were added to the lower chamber, and HUVECs were seeded on the upper chamber. After 24 h of incubation, HUVEC migration was assessed by counting the cells on the lower surface of the membrane; from left to right, the lanes show Ctrl, Sh and Sh/ISO. Scale bar, 100 μm. **i** HUVECs were incubated with CMs from A549 and NCI-H1650 cells in 48-well plates, and their tube formation abilities were evaluated based on the number of tubes per field

To understand the underlying mechanisms and identify the pathways driven by EHD1 in tumor angiogenesis, we analyzed microarray data using classical pathway enrichment analysis. The knockdown of EHD1 inhibited the β-adrenergic signaling pathway, which is involved in tumor angiogenesis and VEGFA regulation (Fig. 3a, Additional file 9: Table S5). More specifically, signaling through β2AR has been shown to play a key role in the control of angiogenesis [20]. According to the data obtained from the TCGA database, EHD1 expression was positively correlated with β2AR expression (Additional file 10: Figure S5a) and the expression of the downstream gene hypoxia-inducible factor (HIF)-1α (Additional file 10: Figure S5b) in lung squamous cell carcinomas and lung adenocarcinoma in the TCGA cohort. The correlation of EHD1 and β2AR in NSCLC cell lines was also confirmed by Western blotting (Fig. 3b).

These results prompted us to examine the effect of EHD1 on the regulation of β2AR in NSCLC. A Western blotting analysis revealed that β2AR expression was significantly lower in EHD1-knockdown cells (Fig. 3c) but higher in EHD1-overexpressing cells (Fig. 3d) compared with the control cells. Interestingly, EHD1 knockdown significantly decreased β2AR protein but not mRNA expression (Additional file 11: Figure S6). These data clearly indicate that EHD1 regulates β2AR expression at the posttranscriptional level but not at the transcriptional level. Conversely, a β2AR agonist (isoproterenol, ISO) or a highly selective β2AR antagonist (ICI118,551, ICI) did not alter EHD1 expression (Additional file 12: Figure S7). Taken together, the results indicate that EHD1 is a critical factor controlling β2AR expression, that β2AR has no impact on EHD1, and that EHD1 activates β2AR signaling in NSCLC.

### EHD1 induces VEGFA expression and angiogenesis in a β2AR-dependent manner in NSCLC

We subsequently tested whether β2AR is necessary for EHD1-induced VEGFA expression and angiogenesis by treating Sh with ISO, an β2AR agonist. As shown in Fig. 3e and f, ISO reversed the decrease in VEGFA expression observed after EHD1 knockdown in NSCLC cells. To explore the role of β2AR in EHD1-induced angiogenesis, HUVECs were incubated with Ctrl-CM, Sh-CM or Sh-CM + ISO, and the results showed that treatment with ISO rescued the Sh/CM-mediated decrease in HUVEC proliferation, migration and tube formation (Fig. 3g-i).

We subsequently used ICI, a highly selective β2AR antagonist. As demonstrated by Western blotting and ELISA, the overexpression of EHD1 increased VEGFA expression, and ICI eliminated this effect (Additional file 13: Figure S8a, b). In vitro angiogenesis assays showed that the viability of HUVECs treated with Sh/R-CM + ICI was significantly lower than that of HUVECs treated with Sh/R-CM (Fig. 8c). Similar results were also obtained in the HUVEC migration and tube formation assays (Additional file 13: Figure S8d, e). These data suggest that β2AR is critical for EHD1-induced VEGFA expression and is required for EHD1-stimulated angiogenesis.

### EHD1 knockdown results in impaired β2AR endocytic recycling

**Fig. 4:**
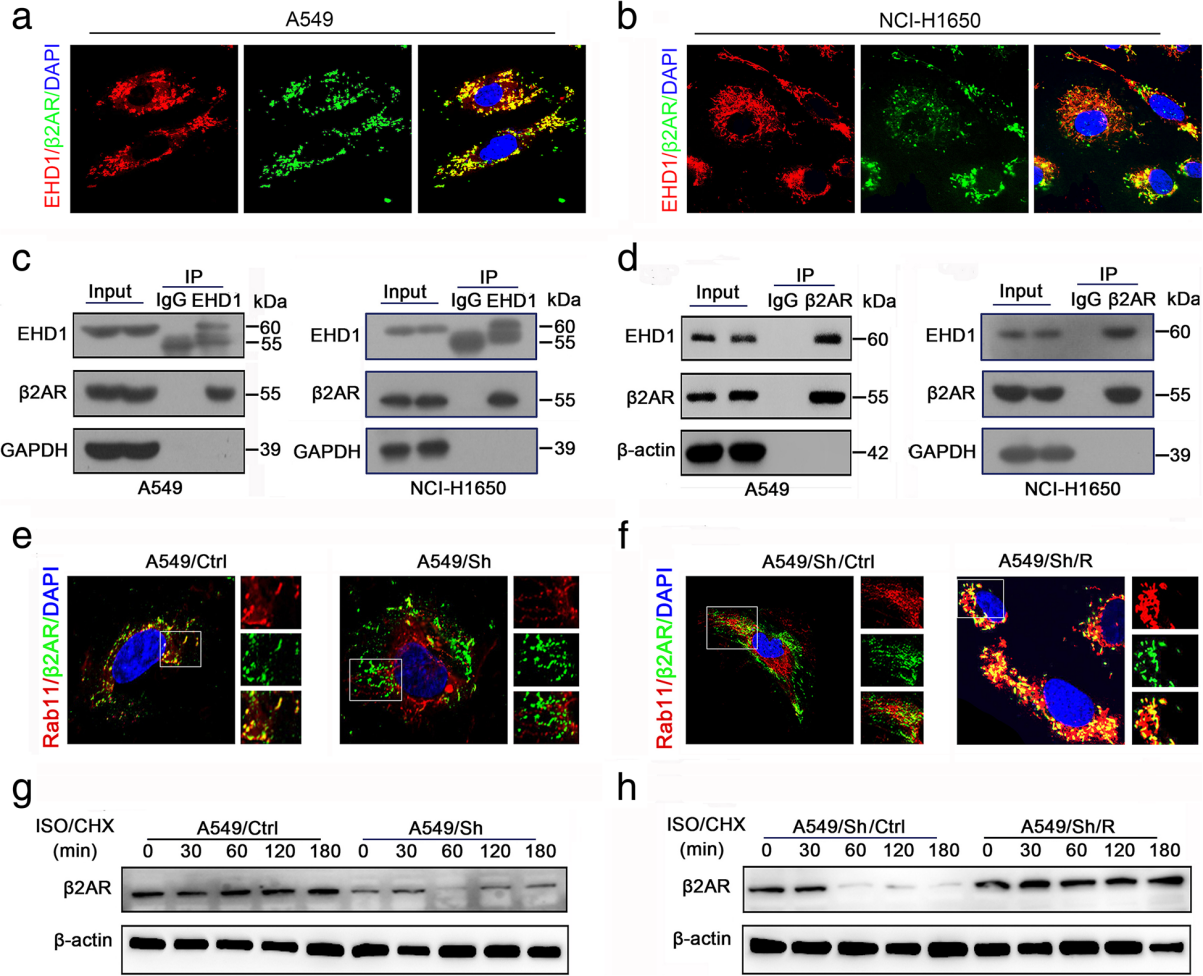
Knockdown of EHD1 suppresses β2AR endocytic recycling. **a, b** Representative images of the colocalization of EHD1 (red) and β2AR (green) in A549 (**a**) and NCI-H1650 (**b**) cells. **c** A549 and NCI-H1650 cell lysates were immunoprecipitated with the anti-EHD1 antibody, and the immunoprecipitates were subjected to Western blot analysis. **d** A549 and NCI-H1650 cell lysates were immunoprecipitated with the anti-β2AR antibody, and the immunoprecipitates were subjected to Western blot analysis. **e, f** Representative images and colocalization of β2AR and Rab11 in A549 and A549/Sh cells (**e**) and in A549/Sh/Ctrl and A549/Sh/R cells (**f**). β2AR was visualized using an anti-β2AR antibody (green fluorescence), Rab11 was visualized using an anti-Rab11 antibody (red fluorescence), and their colocalization is shown in the right-hand panels (merge; yellow fluorescence). Scale bars, 10 μm. **g, h** EHD1 stabilizes β2AR. A549 cells were incubated in serum-free 1640 medium for 16 h and then treated with ISO (10 μm) for the indicated times in the presence of cycloheximide (CHX, 20 μg/ml). The cell lysates were analyzed by Western blot

Our observations demonstrated that EHD1 positively regulates β2AR protein expression but not mRNA expression (Additional file 11: Figure S6). Given that EHD1 plays a critical role in receptor endocytosis and recycling, we speculated that EHD1 regulates β2AR protein via a recycling route through the endocytic pathway. To test this hypothesis, we validated the existence of an interaction between EHD1 and β2AR. We first stained NSCLC cells with anti-EHD1 and anti-β2AR antibodies and observed the colocalization of EHD1 and β2AR protein by confocal microscopy (Fig. 4a, b). The interaction between EHD1 and β2AR protein was further confirmed by coimmunoprecipitation (Co-IP) assays, which demonstrated that endogenous EHD1 colocalized with β2AR (Fig. 4c) and that EHD1 was pulled down by endogenous β2AR (Fig. 4d).

Rab11, which is a recycling endosome marker, interacts with EHD1 through NPF-EH domain interactions and plays a role in recycling from the endocytic recycling compartment [29]. We thus subsequently investigated the colocalization of β2AR and Rab11. A confocal microscopy analysis revealed that the colocalization of β2AR and Rab11 in NSCLC cells was significantly decreased by EHD1 knockdown (Fig. 4e). In contrast, this decrease in β2AR-Rab11 colocalization was reversed by EHD1 reexpression (Fig. 4f).

Once internalized, the receptors are either recycled back to the plasma membrane or sent to late endosomes and on to the lysosomal pathway for degradation (signal termination) [30]. Cycloheximide chase experiments were performed to analyze the role of EHD1 in β2AR stabilization. The half-life of β2AR in EHD1-knockdown cells was markedly shorter than that in control cells, which suggested that in the absence of EHD1, lysosomal delivery is dominant, resulting in β2AR protein degradation (Fig. 4g, h). Altogether, these data suggest that EHD1 enhances the endocytic recycling of β2AR and inhibits the degradation of this receptor.

### EHD1 promotes tumor growth and angiogenesis in vivo

To further investigate the role of EHD1 in NSCLC angiogenesis in vivo, we established xenograft models using nonobese diabetic (NOD)-severe combined immunodeficient (SCID) mice. A549 cells stably transfected with EHD1-Ctrl (Ctrl), EHD1-shRNA (Sh), EHD1-shRNA/Ctrl (Sh/Ctrl) and EHD1-shRNA/R (Sh/R) were subcutaneously injected into the alar skin of the mice, and the tumor growth over 7 and 28 days after implantation was monitored. The mice were sacrificed 28 days after implantation, and the tumors were removed for further analysis.

**Fig. 5:**
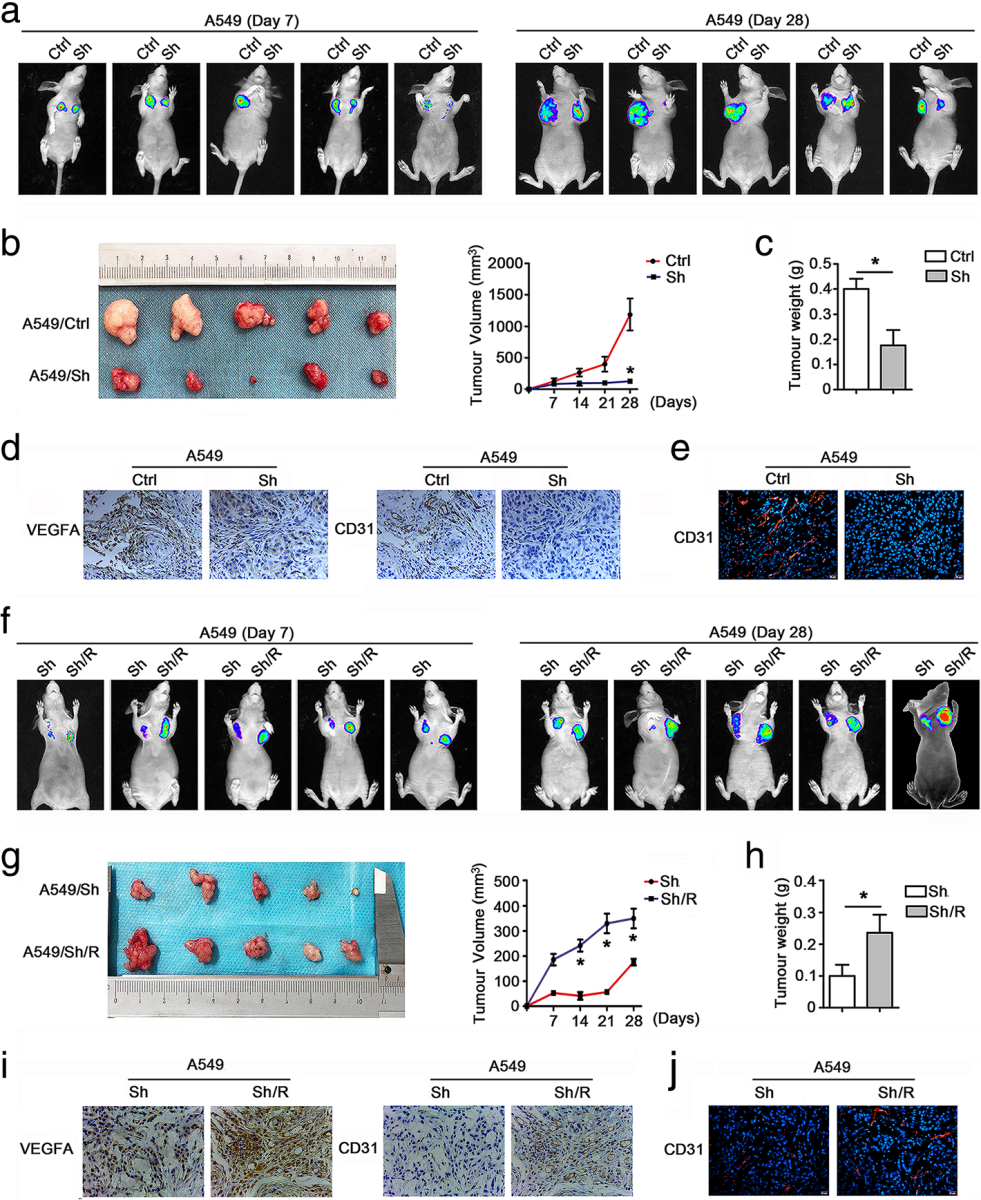
EHD1 contributes to tumor growth and angiogenesis in vivo. **a-e** A549 luciferase cells stably expressing control ShRNA or EHD1 ShRNA were injected into the alar skin of nude mice, and images of the resulting tumors in the mice on days 7 and 28 are shown (**a**). A photograph of the tumors at day 28 post injection is shown (left), and the tumor growth curves of the mice on days 7 to 28 are presented (right) (**b**). The tumor weights were measured, and the data are presented as the means±SDs (*n* = 5, each) (**c**). Representative images of IHC staining for VEGFA and CD31 in xenograft tumor tissues are presented (**d**). Representative immunofluorescent staining of CD31 is shown (**e**). **f-i** A549-luciferase cells stably expressing EHD1 ShRNA or EHD1 ShRNA/R were injected into the alar skin of nude mice. Images of the resulting tumors in the mice on days 7 and 28 are shown (**f**). A photograph of the tumors at day 28 postinjection is shown (left), and the tumor growth curves of the mice on days 7 to 28 are presented (right) (**g**). The tumor weights were measured, and the data are presented as the means±SDs (n = 5, each) (**h**). Representative images of IHC staining for VEGFA and CD31 in xenograft tumor tissues are presented (**i**). Representative immunofluorescent staining of CD31 is shown (**j**). *p < 0.05

Luciferin-based bioluminescence imaging at 7 and 28 days revealed that the knockdown of EHD1 significantly decreased the tumor development potential of A549 cells (Fig. 5a). Relative to the those implanted with the control cells, the mice implanted with EHD1-knockdown cells showed significantly less tumor growth and a decreased tumor weight (Fig. 5b-c). IHC and immunohistofluorescence analyses demonstrated that the expression of CD31, a marker of the MVD, was lower in the tumor tissue obtained with the Sh than in that obtained with the Ctrl (Fig. 5d-e). Conversely, the tumors formed from the Sh/R showed higher luciferase activity (Fig. 5f), larger volumes (Fig. 5g) and heavier weight (Fig. 5h) than those obtained from the Sh/Ctrl. In addition, increased CD31 staining was detected in the tumor tissue obtained from Sh/R compared with that obtained from Sh/Ctrl (Fig. 5i, j). Taken together, these data demonstrate that EHD1 plays an important role in promoting angiogenesis and tumor growth.

### Targeting VEGFA represses EHD1-induced tumor growth and angiogenesis in vivo

**Fig. 6:**
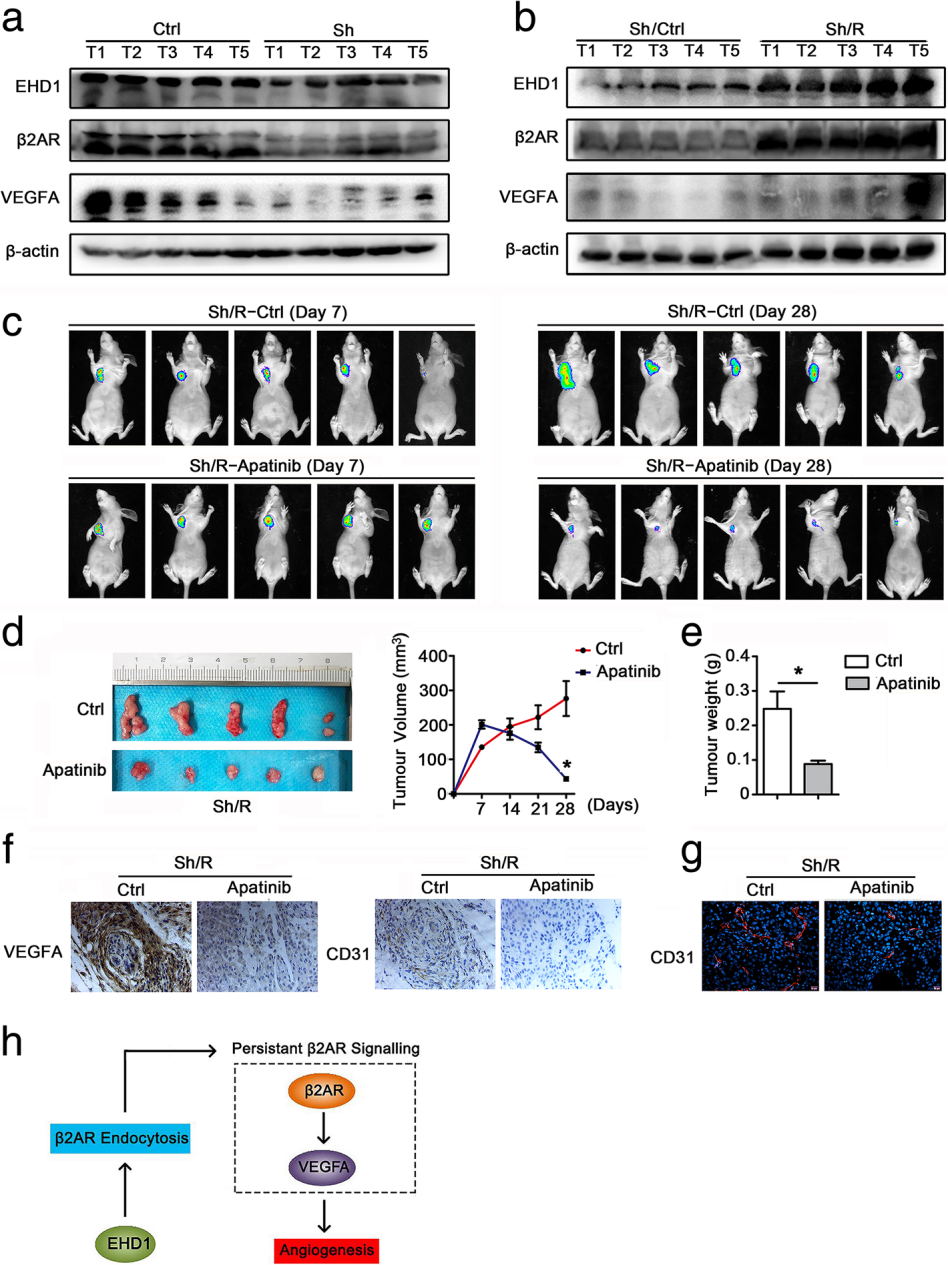
Targeting of VEGFA represses EHD1-induced tumor growth and angiogenesis in vivo. **a-b** The expression of EHD1, β2AR and VEGFA in freshly frozen xenograft tumor tissue was analyzed by Western blot. **c-g** A549 luciferase cells stably expressing EHD1 ShRNA/R-Ctrl or EHD1 ShRNA/R-apatinib were transplanted into the alar skin of nude mice. Images of the resulting tumors in the mice on days 7 and 28 are shown (**c**). A photograph of the tumors on day 28 is shown (left), and the tumor growth curves of the mice on days 7 to 28 are presented (right) (**d**). The tumor weights were measured, and the data are presented as the means±SDs (n = 5, each) (**e**). Representative images of IHC staining for VEGFA and CD31 in xenograft tumor tissues are presented (**f**). Representative immunofluorescent staining of CD31 is shown (**g**). **h** Model of EHD1-induced β2AR/VEGFA expression and angiogenesis in NSCLC

In addition, we investigated the impact of β2AR signaling on EHD1-induced NSCLC angiogenesis and tumor growth in vivo. We detected the expression of EHD1, β2AR and VEGFA in freshly frozen xenograft tumor tissue by Western blot and found that the EHD1 levels were positively correlated with β2AR and VEGFA expression (Fig. 6a, b). Furthermore, once the tumors reached a volume of ~ 100 mm^3^, the mice transplanted with Sh/R were randomly divided into two groups: 1) Sh/R-Ctrl and 2) Sh/R-apatinib. The Sh/R-apatinib mice showed a decreased tumor volume and a lower tumor weight than the control mice, which suggested that VEGFA inhibition impaired the EHD1-induced effect on tumor growth (Fig. 6c-e). Importantly, the IHF results confirmed the decreased expression of CD31 protein detected by IHC analysis in the xenograft tumor tissues of the Sh/R-apatinib group (Fig. 6f, g). These data suggest that targeting β2AR signaling blocks EHD1-induced tumor growth and angiogenesis in vivo.

### EHD1, β2AR, VEGFA and CD31 are coordinately overexpressed in clinical NSCLC specimens

To further examine the relationship between EHD1 and angiogenesis in human NSCLC, we performed IHC staining of EHD1, β2AR, VEGFA and CD31 in 96 NSCLC patient specimens. Consistent with our observations in tumor cell lines and xenograft models, the distribution and intensity of EHD1 were positively correlated with β2AR, VEGFA and CD31 in NSCLC tissue specimens (Additional file 14: Figure S9a). EHD1 was highly expressed in 40.6% of NSCLC cases (*n* = 96). Moreover, the patients with high EHD1 expression also showed high β2AR (Additional file 14: Figure S9b), VEGFA (Additional file 14: Figure S9c) and CD31 expression (Additional file 14: Figure S9d). The intratumoral MVD is used to examine the role of vascularization within the malignant process [31], and thus, MVD scores were calculated by counting the numbers of CD31-positive vessels in whole tumor cross-sections [32]. This analysis revealed that the EHD1 protein levels were positively correlated with the MVD scores (Additional file 14: Figure S9e), which clearly indicated that high EHD1 expression was associated with elevated angiogenesis in NSCLC patients.

## Discussion

In the present study, we provide the first demonstration that EHD1 potentiates tumor angiogenesis in vitro and in vivo. Angiogenesis is considered a cancer progressive factor in tumor growth and metastasis, and antiangiogenic therapy is thought to be an effective therapeutic approach that achieves the expected outcome in patients with cancer [3, 33]. Our findings suggest that EHD1 represents a promising target for antiangiogenetic lung cancer treatment. We and other researchers have reported that EHD1 plays a significant tumor-promoting role as an oncogene in various cancers [8, 11]. Our previous study revealed that EHD1 promotes lung cancer metastasis by inducing epithelial-mesenchymal transition [13]. Given the impact of angiogenesis in cancer metastasis, we will address whether angiogenesis is required for EHD1-induced cancer metastasis in the future.

Here, we confirmed that EHD1 induced VEGFA expression and increased VEGFA secretion. Indeed, VEGFA is the master mediator of tumor angiogenesis and stimulates the migration and proliferation of cultured endothelial cells in different cancer types [34, 35]. The angiogenic function of VEGFA is primarily achieved by binding to receptors, predominantly VEGFR2, on endothelial cells [27]. The binding of VEGF to VEGFR2 induces changes in a variety of downstream signaling pathways, particularly the PI3K/AKT, mitogen-activated protein kinase kinase (MEK)/Erk and P38-mitogen-activated protein kinase (MAPK) pathways, and thereby affects the biological function of endothelial cells [28]. In line with these studies, our results indicated that EHD1-induced VEGFA led to high PI3K/AKT and MEK/Erk signaling pathway activity in endothelial cells.

Moreover, we revealed that EHD1 promoted angiogenesis and tumor growth in a VEGFA-dependent manner. Agents that selectively target VEGFA, its receptor or its downstream signaling pathway effectively improve the survival rates of patients with a variety of cancers [33]. Ovarian cancer patients with EHD1 overexpression exhibit significantly worse responses to bevacizumab, which targets VEGFA [12]. Therefore, we need a large number of NSCLC patients treated with apatinib to analyze the association between the expression of EHD1 and the clinical prognosis of apatinib-treated patients and to further clarify the guiding role of EHD1 in clinical antiangiogenic therapy. In addition, our results will help improve strategies for the selection of NSCLC patients who may particularly benefit from agents that selectively target the VEGFA pathway.

Our current study showed that EHD1 potentiates angiogenesis and tumor growth via the β2AR signaling pathway. A large body of evidence suggests that β2AR signaling activation upregulates the expression of VEGFA and promotes angiogenesis and tumor growth [20, 22]. In our study, although the TCGA data showed that EHD1 was positively correlated with β2AR mRNA expression in NSCLC, the in vitro experiment results showed that EHD1 did not positively regulate β2AR mRNA expression and that the aberrant β2AR signaling pathway did not regulate EHD1 expression. Thus, there might be a co-responsive relationship between EHD1 and β2AR in which EHD1 and β2AR respond to other molecular changes, such as the same transcription factor. In future work, we would like to explore the relationship between EHD1 and β2AR at the transcriptional level.

Moreover, we have indicated that EHD1 governs β2AR signaling by regulating the recycling of β2AR from the endocytic recycling compartment to the plasma membrane. β2AR, as a seven-transmembrane G protein-coupled receptor, undergoes internalization and is then transported to the endosome, from where it is either transported to the late endosome for degradation or transported to the recycling endosome for recovery and transport to the cell surface for persistent activation [30]. Endocytosis trafficking plays a key role in controlling the activity of β2AR [36]. A large body of evidence from many investigators supports the notion that β2AR plays a carcinogenic role dependent on receptor endocytosis [37].

EHD1 regulates the endocytic recycling of transmembrane receptors, such as epithelial growth factor receptor, insulin-like growth factor receptor and colony-stimulating factor-1 receptor [8–10]. Our data reveal a novel function of EHD1 as a regulator of β2AR recycling and demonstrate a requirement for EHD1 in β2AR-mediated downstream functions. Despite its role in endocytic recycling, EHD1 also plays a role in the transport of newly synthesized receptors from the Golgi to the cell surface [10]. Future studies in our laboratory will test this function of EHD1.

Here, we propose a working model of EHD1 function in tumor angiogenesis. The coupling of β2AR with its ligands induces the internalization of β2AR, which is then delivered to early endosomes. EHD1 promotes the endocytic recycling of β2AR, resulting in an increased amount of β2AR available for oncogenic signaling. Thus, EHD1 overexpression leads to persistent β2AR signaling activity, and upregulated VEGFA stimulates tumor angiogenesis (Fig. 6h).

## Conclusion

Taken together, the results obtained in this study reveal that EHD1 potentiates NSCLC growth and angiogenesis through the β2AR/VEGFA signaling pathway both in vitro and in vivo. Moreover, EHD1 governs β2AR signaling by promoting the endocytic recycling of β2AR. These observations improve our understanding of how EHD1 impacts cancer development and progression and provide new insights into the underlying mechanisms of NSCLC angiogenesis, which emphasize that EHD1 is a potential antiangiogenic therapeutic target in NSCLC.

## Additional files

Additional file 1: **Table S1.** Association between EHD1 expression and clinicopathological characteristics of NSCLC patients. (DOC 63 kb)

Additional file 2: **Figure S1.** EHD1 overexpression is associated with poor prognosis. a, b Kaplan-Meier curves of overall survival (a) and disease-free survival (b) for patients with NSCLC from Harbin Medical University Cancer Center (HMUCC). c, d Kaplan-Meier curves for overall survival (c) and disease-free survival (d) for patients with NSCLC in the TCGA database. e Kaplan-Meier overall survival curves for cancer patients with low EHD1 expression (*n* = 5037) and high EHD1 expression (*n* = 5039; *p* = 0.0018). f Kaplan-Meier progression-free interval curves for cancer patients with low (*n* = 5118) and high (*n* = 5117) expression of EHD1 (*p* < 0.0001). (TIF 4499 kb)

Additional file 3: The association between EHD1 and tumor angiogenesis. (XLSX 59 kb)

Additional file 4: The association between EHD1 and vascular endothelial cell proliferation. (XLSX 25 kb)

Additional file 5: The association between EHD1 and vascular endothelial cell migration. (XLSX 26 kb)

Additional file 6: **Figure S2.** Reexpression of EHD1 promotes angiogenesis. a Western blot analysis of EHD1 expression in A549 and NCI-H1650 cells after EHD1 reexpression. b The viability of HUVECs was detected by CCK8 assay. HUVECs were incubated in 96-well plates with CMs from A549 and NCI-H1650 cells. c CMs were added to the lower chamber, and HUVECs were seeded on the upper chamber. After 24 h of incubation, HUVEC migration was assessed by counting the cells on the lower surface of the membrane; from left to right, the lanes show Sh/UT, Sh/Ctrl and Sh/R. Scale bar, 100 μm. d HUVECs were incubated in 48-well plates with CMs from A549 and NCI-H1650 cells, and their tube formation abilities were evaluated based on the number of tubes per field. *p < 0.05; **p < 0.01. (TIF 10271 kb)

Additional file 7: **Figure S3.** EHD1 knockdown attenuates p-AKT and p-Erk expression in HUVECs. p-AKT and p-Erk expression in HUVECs cocultured with Sh was significantly attenuated compared with that in HUVECs cocultured with Ctrl. (TIF 8001 kb)

Additional file 8: **Figure S4.** Reexpression of EHD1 increases VEGFA expression and angiogenesis. a Western blot analysis of VEGFA expression in A549 and NCI-H1650 cells after EHD1 reexpression. β-actin served as the loading control. b A549 and NCI-H1650 cells were incubated overnight with serum-free1640 medium, and their CMs were used to detect the level of VEGFA secretion; from left to right, the lanes show Sh/Ctrl and Sh/R, respectively. **c** CMs were added to the lower chamber, and HUVECs were seeded on the upper chamber. After 24 h of incubation, HUVEC migration was assessed by counting the cells on the lower surface of the membrane. Scale bar, 100 μm. d HUVECs were incubated in 48-well plates with CMs from A549 and NCI-H1650 cells, and their tube formation abilities were evaluated based on the number of tubes per field. *p < 0.05; **p < 0.01. (TIF 10245 kb)

Additional file 9: The overlap between β2AR-dependent gene expression and EHD1-regulated gene expression. (XLSX 14 kb)

Additional file 10: **Figure S5.** EHD1 expression is positively correlated with β2AR signaling in NSCLC. a Analyses of TCGA lung adenocarcinoma and lung squamous cell carcinoma samples show that EHD1 expression is positively correlated with β2AR expression. b Analyses of TCGA lung adenocarcinoma and lung squamous cell carcinoma samples show that EHD1 expression is positively correlated with HIF1-α expression. (TIF 8835 kb)

Additional file 11: **Figure S6.** EHD1 has no effect on β2AR mRNA expression. qRT-PCR analysis of EHD1 and β2AR mRNA levels in A549 cells; the lanes show Ctrl and Sh. (TIF 880 kb)

Additional file 12: **Figure S7.** EHD1 expression is not affected by activation or inhibition of the β2AR signaling pathway. Western blot analysis of EHD1 expression in A549 cells after EHD1 knockdown and reexpression. A549/Sh cells were treated with ISO for 4 h to activate β2AR signaling, and A549/Sh/R cells were treated with ICI for 4 h to inhibit β2AR signaling activation. (JPG 1514 kb)

Additional file 13: **Figure S8.** Blocking β2AR inhibited EHD1-induced VEGFA expression and angiogenesis. a Western blot analysis of VEGFA expression in A549 cells after EHD1 knockdown and reeexpression; the lanes show Sh/Ctrl, Sh/R, and Sh/R/ICI, respectively. b A549 cells were incubated overnight with serum-free 1640 medium and corresponding reagents, and their CMs were used to detect the level of VEGFA secretion. c The viability of HUVECs was detected by CCK8 assay. d HUVEC migration was assessed by counting the cells on the lower surface of the membrane. Scale bar, 100 μm. e HUVECs were incubated with CMs from A549 cells in 48-well plates, and their tube formation abilities were examined based on the number of tubes per field. *p < 0.05; **p < 0.01. (TIF 8743 kb)

Additional file 14: **Figure S9.** Correlation of EHD1 with β2AR, VEGFA and CD31 expression and angiogenesis in HCC tissues. a Representative IHC images of NSCLC samples with low and high expression of the indicated proteins. Scale bar, 50 μm and 100 μm. b-d The tissue samples were divided into two groups according to the level of EHD1 expression: the low-expression group (scores of 0 and 1) and the high-expression group (scores of 2 and 3). Patients with high EHD1 expression showed high β2AR expression (b), VEGFA expression (**c**), CD31 (d) and MVD (e). e The horizontal lines indicate the intermediate values; the bottom and top of the box represent the 25th and 75th percentiles, respectively; and vertical bars represent the data range. (TIF 9217 kb)

## Abbreviations

CM: conditioned medium
Co-IP: coimmunoprecipitation (Co-IP)
EHD1: Mammalian Eps15 homology domain 1
ELISA: enzyme-linked immunosorbent assay (ELISA)
HUVEC: Human umbilical vein endothelial cell
ICI: ICI118,551
IF: immunofluorescence (IF)
IHC: immunohistochemistry (IHC)
ISO: isoproterenol
NSCLC: non-small cell lung cancer
OD: optical density
OS: overall survival
qRT-PCR: quantitative real-time reverse transcription-polymerase chain reaction
TCGA: The Cancer Genome Atlas
VEGFA: vascular endothelial growth factors A
β2AR: β2-adrenoceptors
