# Supplementary material for: Mammalian Eps15 homology domain 1 potentiates angiogenesis of non-small cell lung cancer by regulating β2AR signaling
Source: J Exp Clin Cancer Res. 2019 Apr 25;38:174. doi: 10.1186/s13046-019-1162-7 (PMC6482525; doi:10.1186/s13046-019-1162-7)
Supplement: Supplementary file 1 — Table S1. Association between EHD1 expression and clinicopathological characteristics of NSCLC patients. (DOC 63 kb) [file 13046_2019_1162_MOESM1_ESM.doc]

Table S1. Association between EHD1 expression and clinicopathological characteristics of NSCLC patients.

|  |  | | | EHD1 expression | | |  |
| --- | --- | --- | --- | --- | --- | --- | --- |
| Variable | All patients | | | High (%) | Low (%) | *P* | |
|  | (*n* = 96) | | | (*n* = 39) | (*n* = 57) |  |  |
|  | | |  |  |  |  | |
| Smoking | |  | |  |  |  | |
| Never | | 46 | | 20 (51) | 26 (46) | 0.585 | |
| Ever | | 50 | | 19 (49) | 31 (54) |  | |
|  | |  | |  |  |  | |
| Gender | |  | |  |  |  | |
| Male | | 62 | | 26 (67) | 36 (63) | 0.724 | |
| Female | | 34 | | 13 (33) | 21 (37) |  | |
|  | |  | |  |  |  | |
| Age (years) | |  | |  |  |  | |
| <60 | | 56 | | 22 (56) | 34 (60) | 0.752 | |
| ≥60 | | 40 | | 17 (44) | 23 (40) |  | |
|  | |  | |  |  |  | |
| Differentiation | |  | |  |  |  | |
| Well | | 15 | | 6 (15) | 9 (16) | 0.890 | |
| Moderate | | 27 | | 10 (26) | 17 (30) |  | |
| Poor | | 54 | | 23 (59) | 31 (54) |  | |
|  | |  | |  |  |  | |
| Histological cell type | |  | |  |  |  | |
| Adenocarcinoma | | 76 | | 29 (74) | 47 (82) | 0.337 | |
| Squamous cell carcinoma | | 20 | | 10 (26) | 10 (18) |  | |
|  | |  | |  |  |  | |
| pStage | |  | |  |  |  | |
| I | | 32 | | 8 (20.5) | 24 (42) | 0.046* | |
| II | | 23 | | 9 (23) | 14 (25) |  | |
| III | | 41 | | 22 (56.5) | 19 (33) |  | |
|  | |  | |  |  |  | |
| pT classification | |  | |  |  |  | |
| T1 | | 29 | | 7 (18) | 22 (38.5) | 0.0358*^#^ | |
| T2 | | 60 | | 27 (69) | 33 (58) |  | |
| T3/4 | | 7 | | 5 (13) | 2 (3.5) |  | |
|  | |  | |  |  |  | |
| Lymph node metastasis | |  | |  |  | 0.302 | |
| Present | | 53 | | 24 (62) | 29 (51) |  | |
| Absent | | 43 | | 15 (38) | 28 (49) |  | |
|  | |  | |  |  |  | |
| Adjuvant therapy | |  | |  |  |  | |
| Yes | | 64 | | 22(56) | 42(74) | 0.186 | |
| No | | 32 | | 17(44) | 15(26) |  | |

Abbreviations: NSCLC = non-small cell lung cancer; pTNM stage = tumor, node, metastasis (pathological stage); pT = pathological T stage; n = number of patients. Ever: smoking at any time from the beginning of life. *p* value: the difference of clinicopathological characteristics between the EHD1 high expression group and low expression group. **p*< 0.05 was considered statistically significant. ^#^ Fisher’s exact test.
