# Supplementary figures and images for: Mammalian Eps15 homology domain 1 potentiates angiogenesis of non-small cell lung cancer by regulating β2AR signaling
Source: J Exp Clin Cancer Res. 2019 Apr 25;38:174. doi: 10.1186/s13046-019-1162-7 (PMC6482525; doi:10.1186/s13046-019-1162-7)

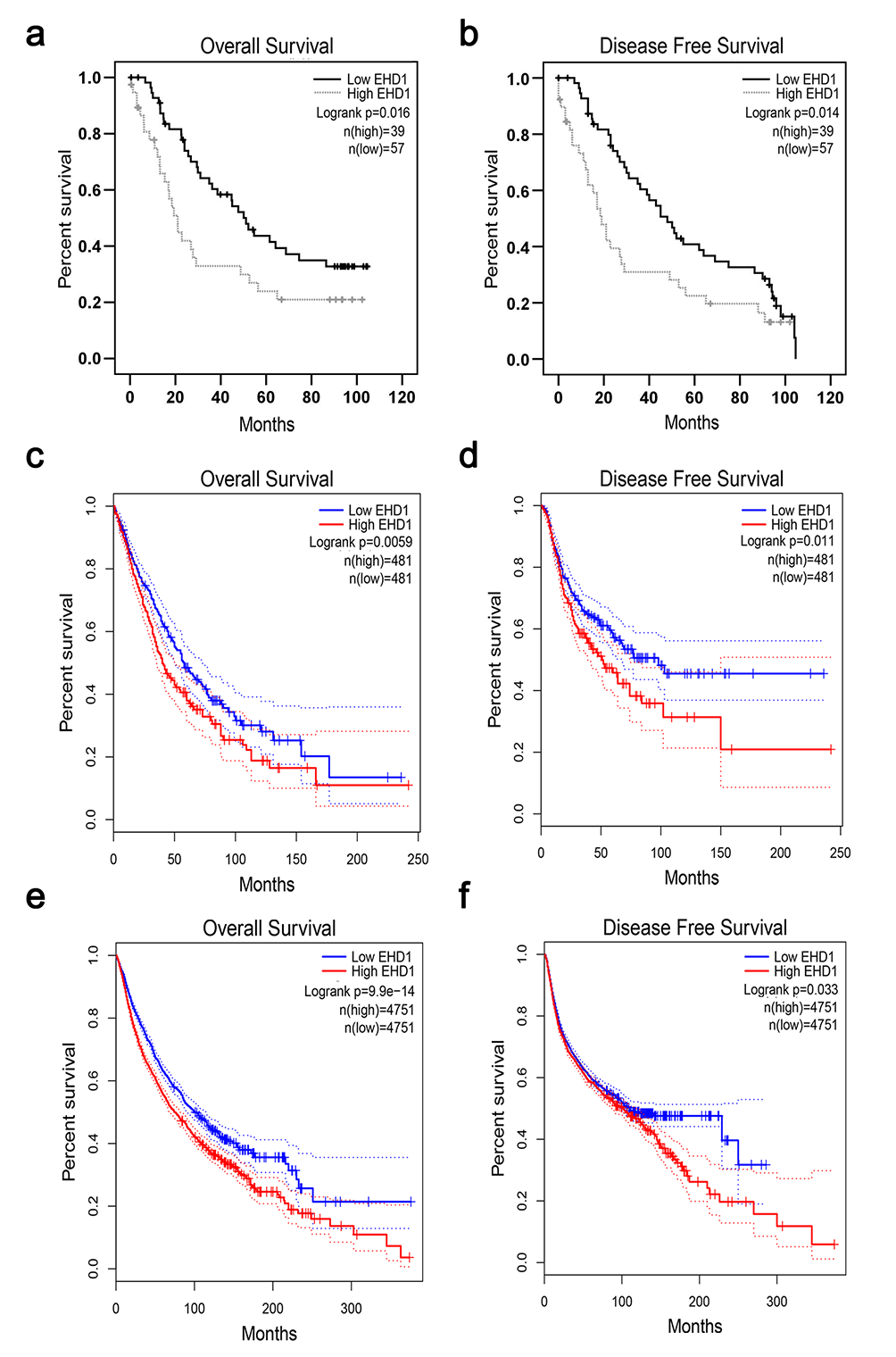

Supplement: Supplementary file 2 — Figure S1. EHD1 overexpression is associated with poor prognosis. a, b Kaplan-Meier curves of overall survival (a) and disease-free survival (b) for patients with NSCLC from Harbin Medical University Cancer Center (HMUCC). c, d Kaplan-Meier curves for overall survival (c) and disease-free survival (d) for patients with NSCLC in the TCGA database. e Kaplan-Meier overall survival curves for cancer patients with low EHD1 expression (n = 5037) and high EHD1 expression (n = 5039; p = 0.0018). f Kaplan-Meier progression-free interval curves for cancer patients with low (n = 5118) and high (n = 5117) expression of EHD1 (p < 0.0001). (TIF 4499 kb) [file 13046_2019_1162_MOESM2_ESM.tif]

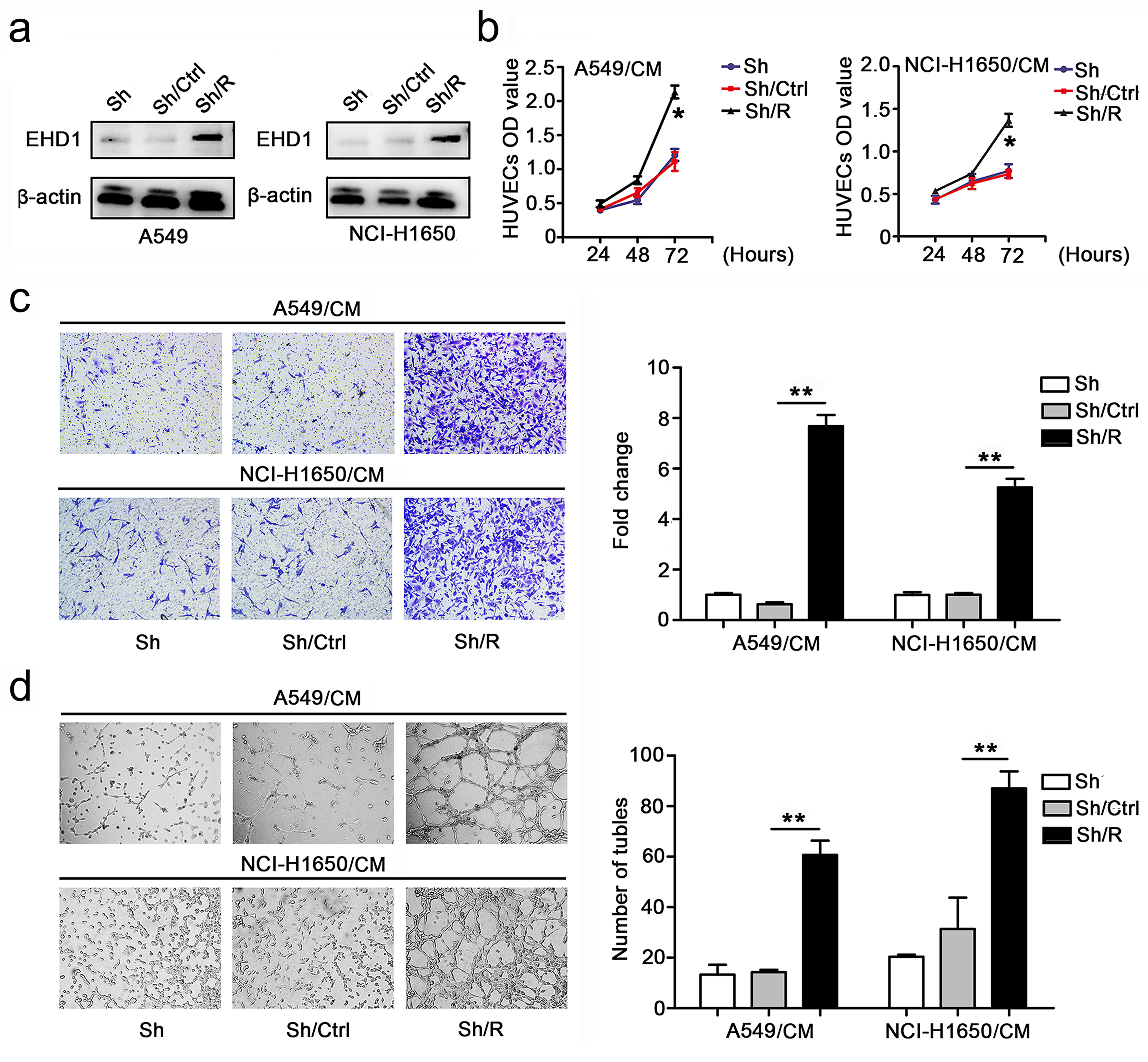

Supplement: Supplementary file 6 — Figure S2. Reexpression of EHD1 promotes angiogenesis. a Western blot analysis of EHD1 expression in A549 and NCI-H1650 cells after EHD1 reexpression. b The viability of HUVECs was detected by CCK8 assay. HUVECs were incubated in 96-well plates with CMs from A549 and NCI-H1650 cells. c CMs were added to the lower chamber, and HUVECs were seeded on the upper chamber. After 24 h of incubation, HUVEC migration was assessed by counting the cells on the lower surface of the membrane; from left to right, the lanes show Sh/UT, Sh/Ctrl and Sh/R. Scale bar, 100 μm. d HUVECs were incubated in 48-well plates with CMs from A549 and NCI-H1650 cells, and their tube formation abilities were evaluated based on the number of tubes per field. *p < 0.05; **p < 0.01. (TIF 10271 kb) [file 13046_2019_1162_MOESM6_ESM.tif]

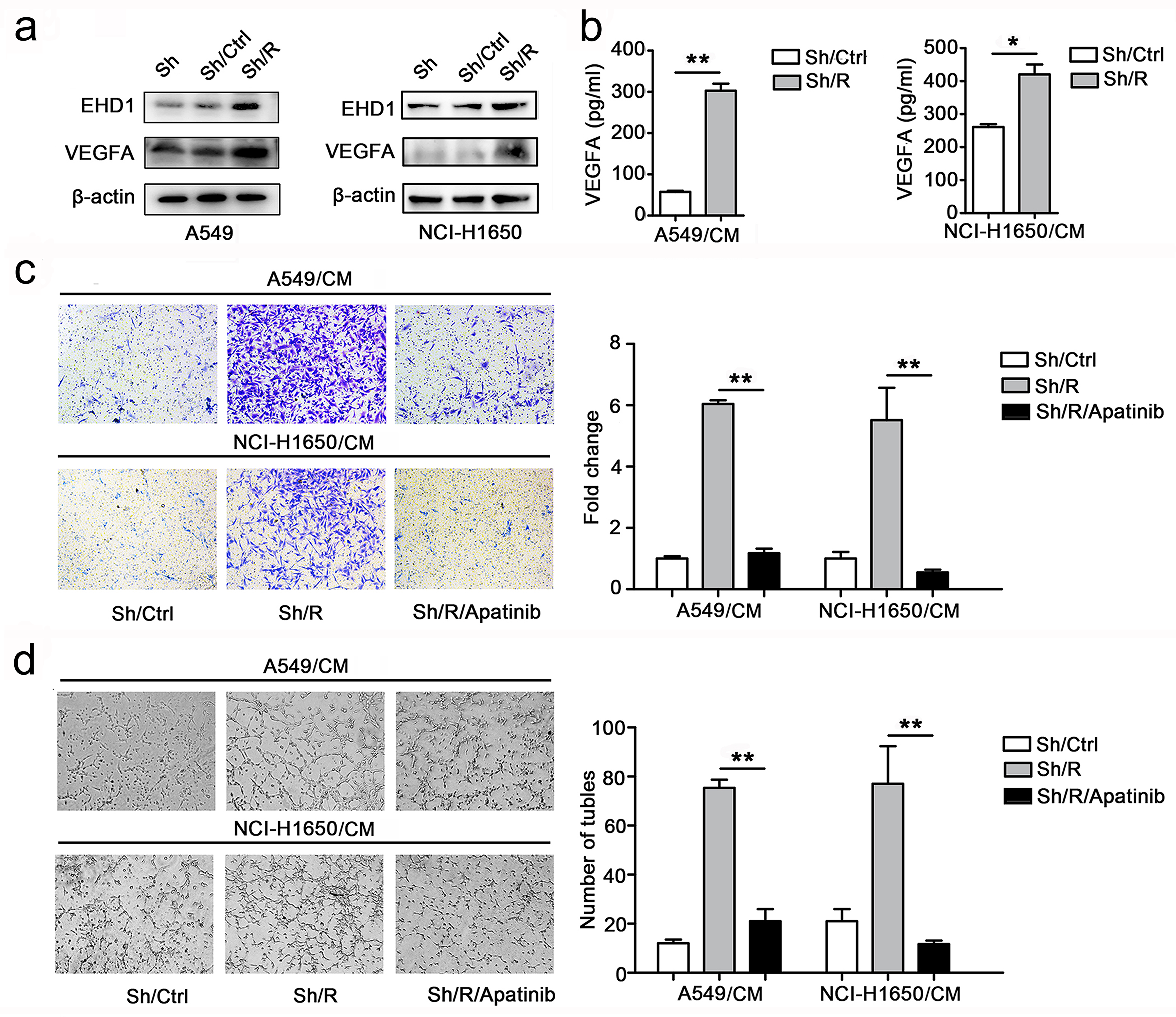

Supplement: Supplementary file 8 — Figure S4. Reexpression of EHD1 increases VEGFA expression and angiogenesis. a Western blot analysis of VEGFA expression in A549 and NCI-H1650 cells after EHD1 reexpression. β-actin served as the loading control. b A549 and NCI-H1650 cells were incubated overnight with serum-free1640 medium, and their CMs were used to detect the level of VEGFA secretion; from left to right, the lanes show Sh/Ctrl and Sh/R, respectively. c CMs were added to the lower chamber, and HUVECs were seeded on the upper chamber. After 24 h of incubation, HUVEC migration was assessed by counting the cells on the lower surface of the membrane. Scale bar, 100 μm. d HUVECs were incubated in 48-well plates with CMs from A549 and NCI-H1650 cells, and their tube formation abilities were evaluated based on the number of tubes per field. *p < 0.05; **p < 0.01. (TIF 10245 kb) [file 13046_2019_1162_MOESM8_ESM.tif]

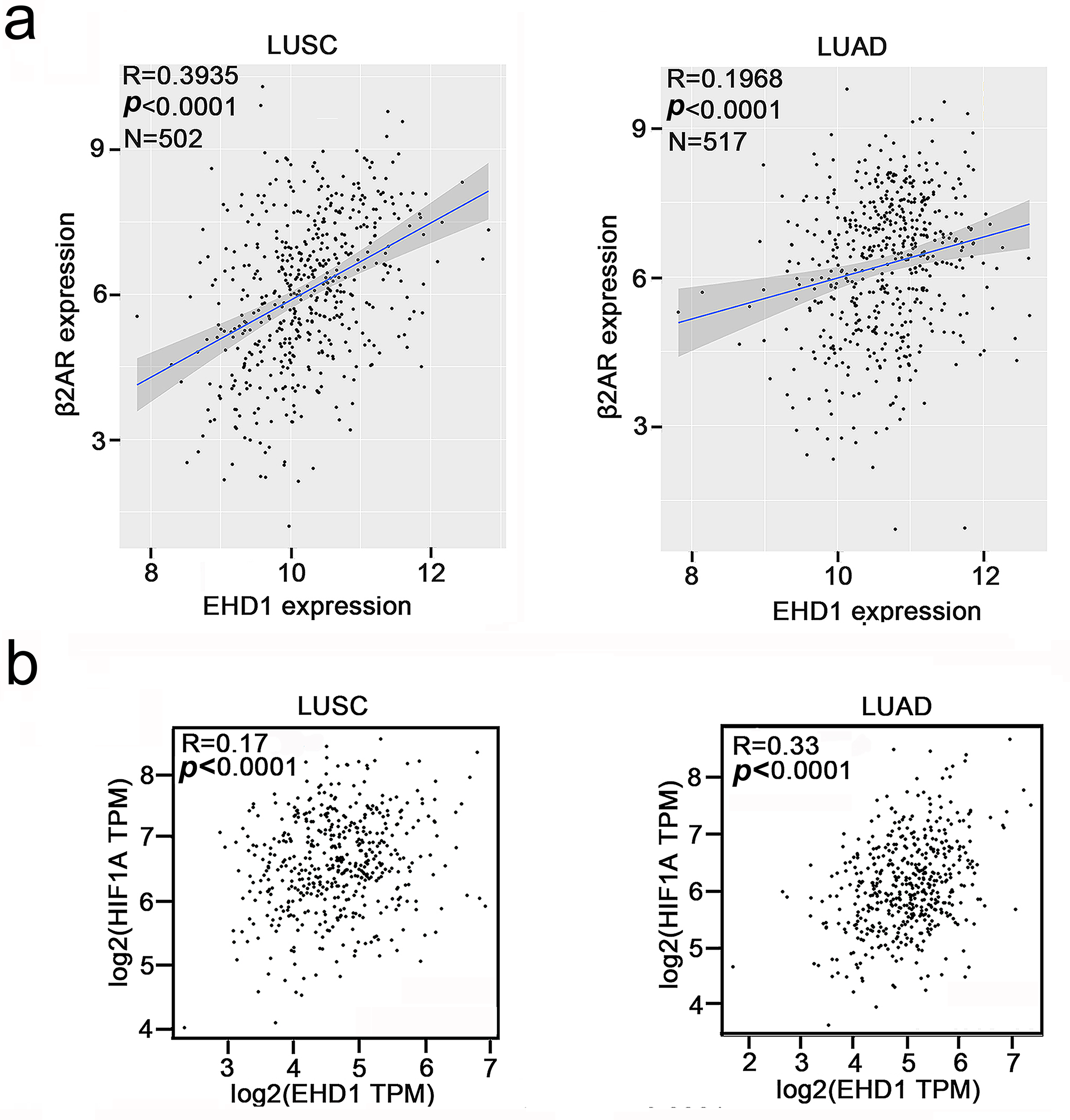

Supplement: Supplementary file 10 — Figure S5. EHD1 expression is positively correlated with β2AR signaling in NSCLC. a Analyses of TCGA lung adenocarcinoma and lung squamous cell carcinoma samples show that EHD1 expression is positively correlated with β2AR expression. b Analyses of TCGA lung adenocarcinoma and lung squamous cell carcinoma samples show that EHD1 expression is positively correlated with HIF1-α expression. (TIF 8835 kb) [file 13046_2019_1162_MOESM10_ESM.tif]

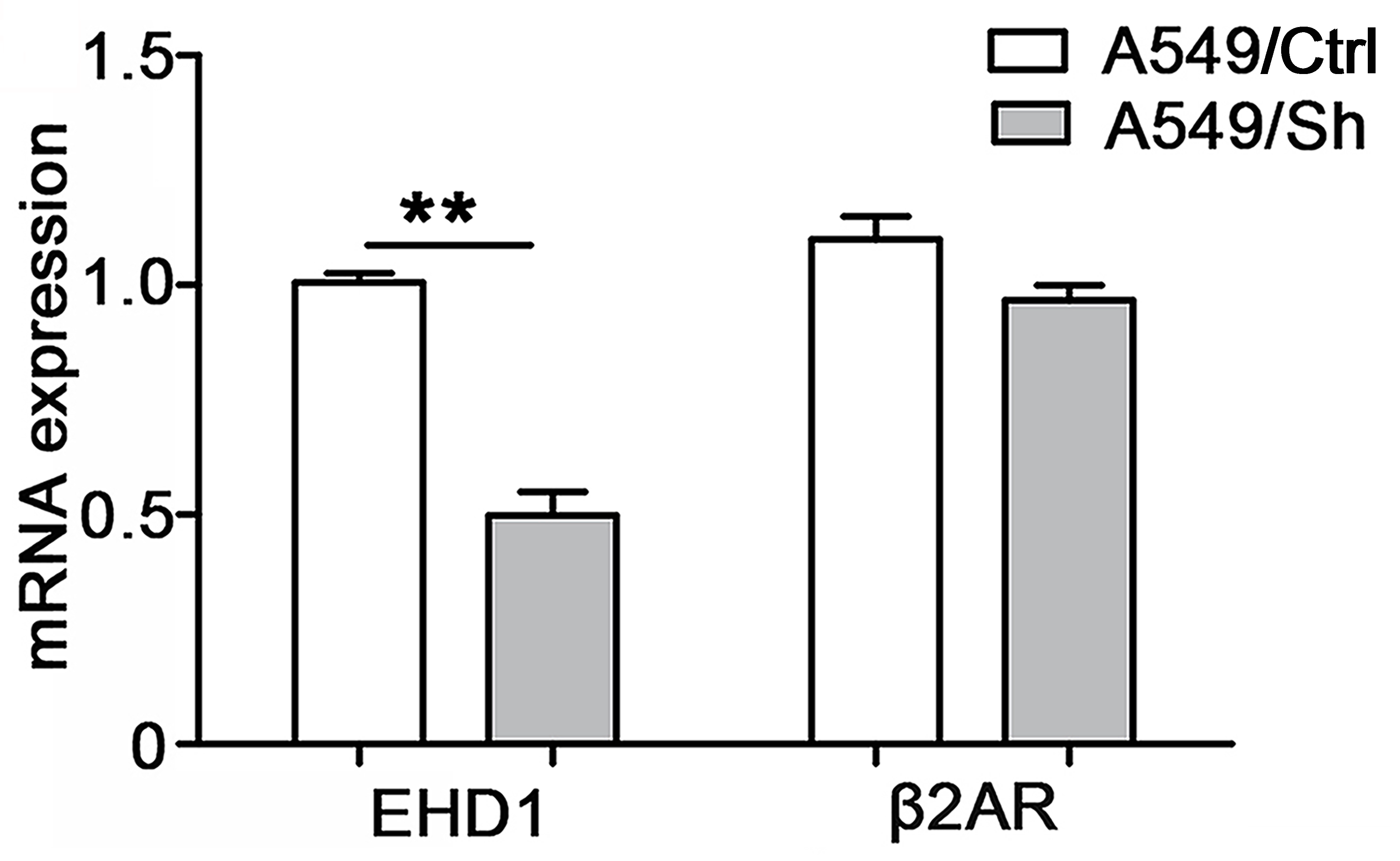

Supplement: Supplementary file 11 — Figure S6. EHD1 has no effect on β2AR mRNA expression. qRT-PCR analysis of EHD1 and β2AR mRNA levels in A549 cells; the lanes show Ctrl and Sh. (TIF 880 kb) [file 13046_2019_1162_MOESM11_ESM.tif]

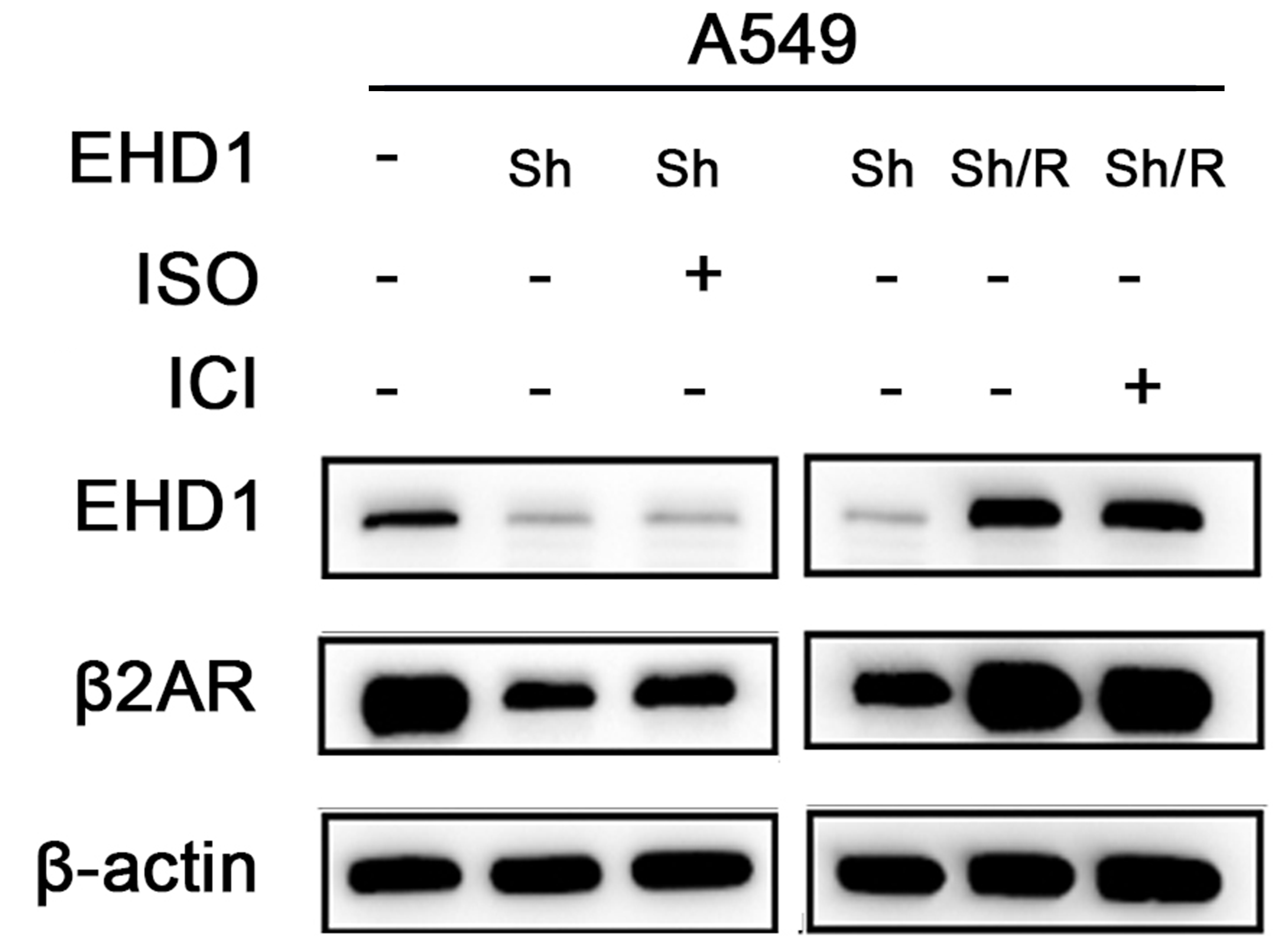

Supplement: Supplementary file 12 — Figure S7. EHD1 expression is not affected by activation or inhibition of the β2AR signaling pathway. Western blot analysis of EHD1 expression in A549 cells after EHD1 knockdown and reexpression. A549/Sh cells were treated with ISO for 4 h to activate β2AR signaling, and A549/Sh/R cells were treated with ICI for 4 h to inhibit β2AR signaling activation. (JPG 1514 kb) [file 13046_2019_1162_MOESM12_ESM.jpg]

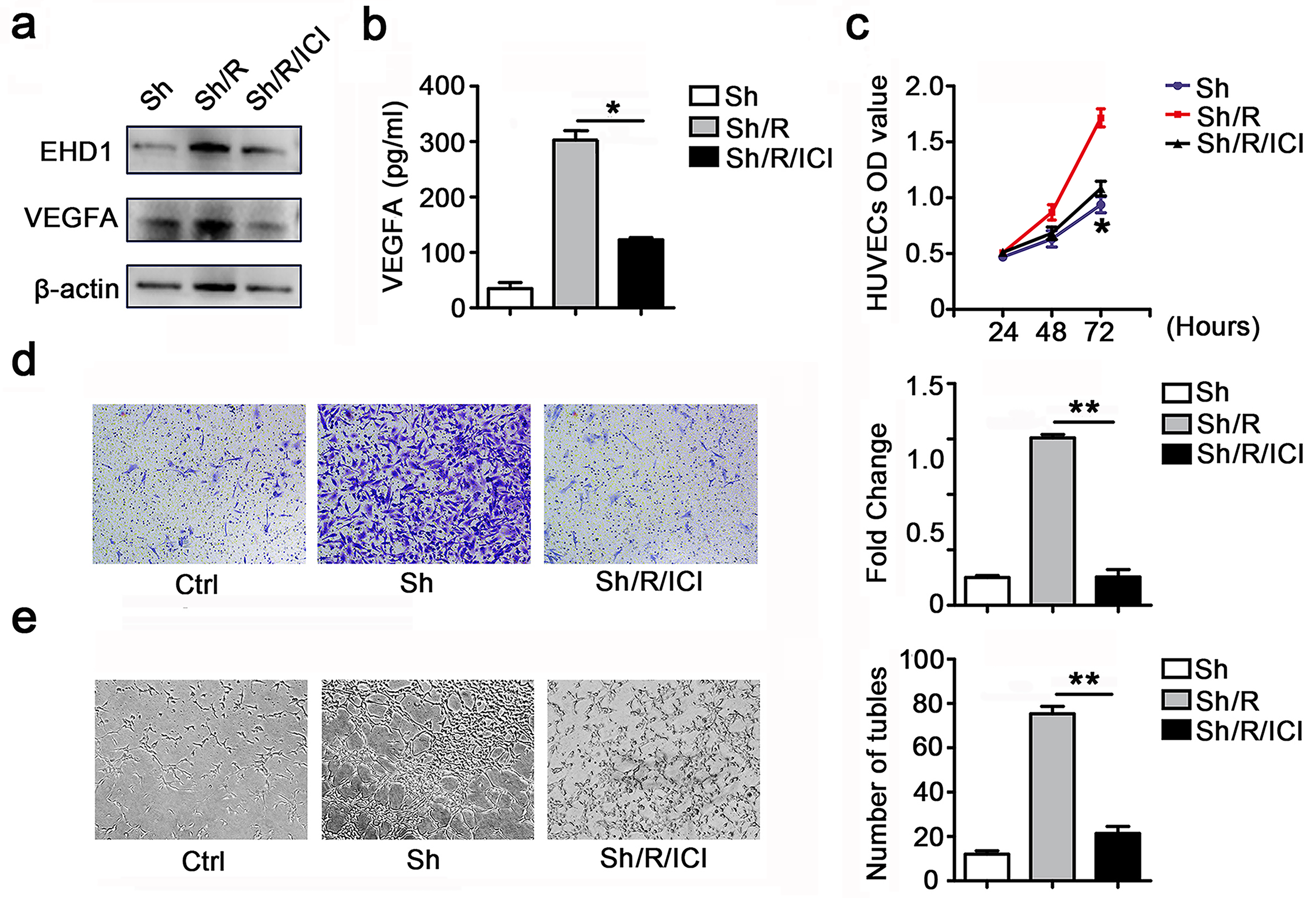

Supplement: Supplementary file 13 — Figure S8. Blocking β2AR inhibited EHD1-induced VEGFA expression and angiogenesis. a Western blot analysis of VEGFA expression in A549 cells after EHD1 knockdown and reeexpression; the lanes show Sh/Ctrl, Sh/R, and Sh/R/ICI, respectively. b A549 cells were incubated overnight with serum-free 1640 medium and corresponding reagents, and their CMs were used to detect the level of VEGFA secretion. c The viability of HUVECs was detected by CCK8 assay. d HUVEC migration was assessed by counting the cells on the lower surface of the membrane. Scale bar, 100 μm. e HUVECs were incubated with CMs from A549 cells in 48-well plates, and their tube formation abilities were examined based on the number of tubes per field. *p < 0.05; **p < 0.01. (TIF 8743 kb) [file 13046_2019_1162_MOESM13_ESM.tif]

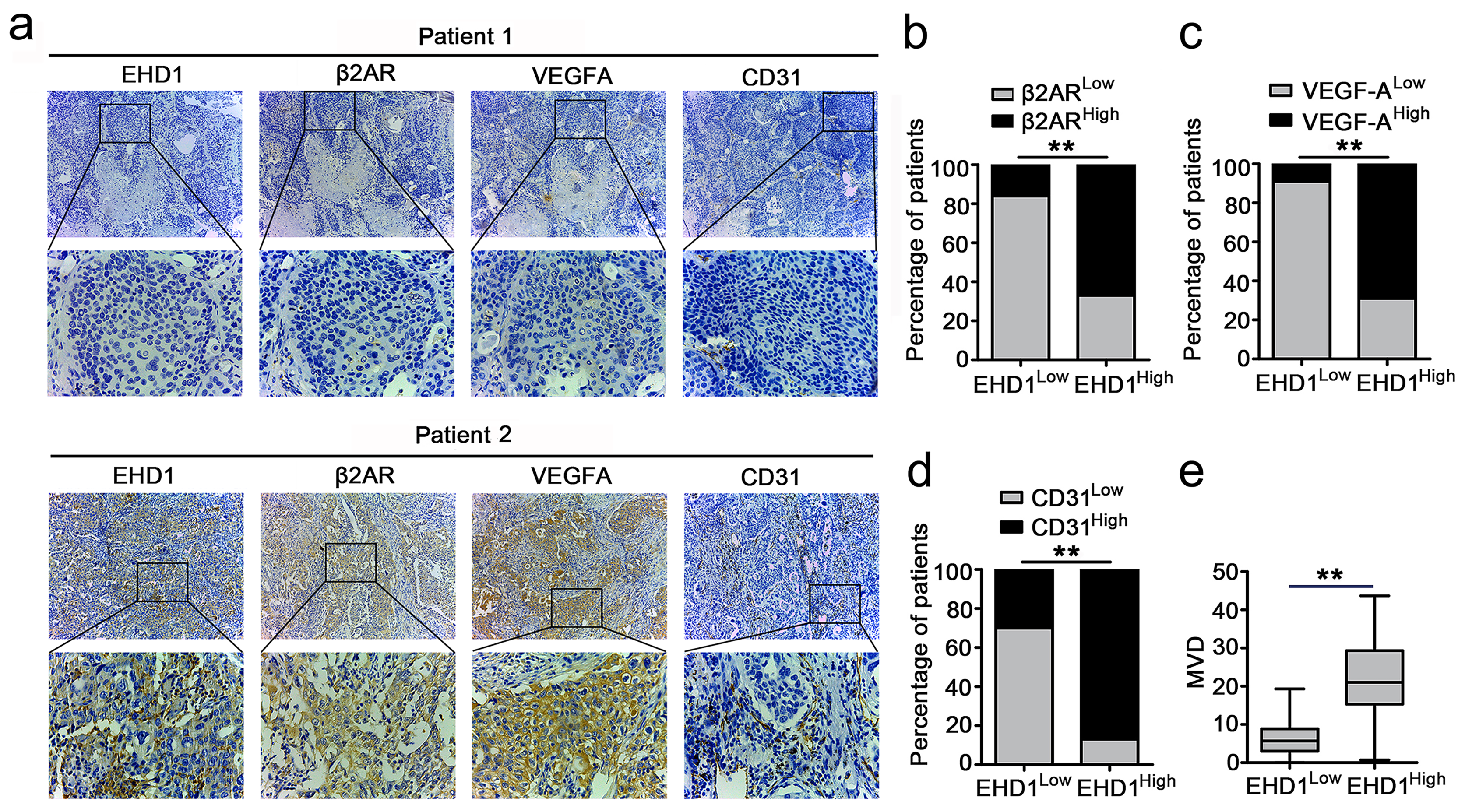

Supplement: Supplementary file 14 — Figure S9. Correlation of EHD1 with β2AR, VEGFA and CD31 expression and angiogenesis in HCC tissues. a Representative IHC images of NSCLC samples with low and high expression of the indicated proteins. Scale bar, 50 μm and 100 μm. b-d The tissue samples were divided into two groups according to the level of EHD1 expression: the low-expression group (scores of 0 and 1) and the high-expression group (scores of 2 and 3). Patients with high EHD1 expression showed high β2AR expression (b), VEGFA expression (c), CD31 (d) and MVD (e). e The horizontal lines indicate the intermediate values; the bottom and top of the box represent the 25th and 75th percentiles, respectively; and vertical bars represent the data range. (TIF 9217 kb) [file 13046_2019_1162_MOESM14_ESM.tif]
